# CD4^+^ T Cell-derived IL-10 Promotes *Brucella abortus* Persistence via Modulation of Macrophage Function

**DOI:** 10.1371/journal.ppat.1003454

**Published:** 2013-06-20

**Authors:** Mariana N. Xavier, Maria G. Winter, Alanna M. Spees, Kim Nguyen, Vidya L. Atluri, Teane M. A. Silva, Andreas J. Bäumler, Werner Müller, Renato L. Santos, Renée M. Tsolis

**Affiliations:** 1 Department of Medical Microbiology and Immunology, School of Medicine, University of California at Davis, Davis, California, United States of America; 2 Departamento de Clínica e Cirurgia Veterinárias, Universidade Federal de Minas Gerais, Belo Horizonte, Minas Gerais, Brazil; 3 Faculty of Life Sciences, University of Manchester, Manchester, United Kingdom; Yale University School of Medicine, United States of America

## Abstract

Evasion of host immune responses is a prerequisite for chronic bacterial diseases; however, the underlying mechanisms are not fully understood. Here, we show that the persistent intracellular pathogen *Brucella abortus* prevents immune activation of macrophages by inducing CD4^+^CD25^+^ T cells to produce the anti-inflammatory cytokine interleukin-10 (IL-10) early during infection. IL-10 receptor (IL-10R) blockage in macrophages resulted in significantly higher NF-kB activation as well as decreased bacterial intracellular survival associated with an inability of *B. abortus* to escape the late endosome compartment *in vitro*. Moreover, either a lack of IL-10 production by T cells or a lack of macrophage responsiveness to this cytokine resulted in an increased ability of mice to control *B. abortus* infection, while inducing elevated production of pro-inflammatory cytokines, which led to severe pathology in liver and spleen of infected mice. Collectively, our results suggest that early IL-10 production by CD25^+^CD4^+^ T cells modulates macrophage function and contributes to an initial balance between pro-inflammatory and anti-inflammatory cytokines that is beneficial to the pathogen, thereby promoting enhanced bacterial survival and persistent infection.

## Author Summary

*Brucella* spp. are pathogens causing chronic intracellular infections that evade detection by pattern recognition receptors of the innate immune system. In this work, we tested the hypothesis that, in addition to eliciting a weak proinflammatory response during infection, induction of an immunoregulatory environment early during infection could promote persistent infection. Our results show that IL-10 produced at early time points is important for blunting inflammatory responses to *B. abortus* in infected tissues. CD4^+^ T cells are an important source of this cytokine, since mice lacking T cell-derived IL-10 exhibited increased inflammatory pathology and also were better able to control *B. abortus* infection. A target of this CD4 T cell-derived IL-10 is macrophages, since treatment of these cells with IL-10 in vitro supported intracellular replication of *B. abortus*, while blocking IL-10 restricted the ability of *B. abortus* to exit the phagolysosomal compartment and replicate intracellularly. Further, mice conditionally deficient for IL-10 receptor on macrophages were better able to control infection with *B. abortus*. Taken together, our results support a model in which IL-10 production by T cells promotes chronic infection by rendering macrophages more permissive for intracellular replication of *B. abortus*.

## Introduction

Persistent bacterial infections have a significant impact on public health [1]. While evasion of host immune responses is a prerequisite for these chronic infections, the underlying mechanisms are not fully understood. Human brucellosis, caused by the intracellular gram-negative coccobacilli *Brucella* spp., is considered one of the most important zoonotic diseases worldwide, with more than 500,000 new human cases reported annually [2]. The disease is characterized by a long incubation period that leads to a chronic, sometimes lifelong, debilitating infection with serious clinical manifestations such as fever, arthritis, hepatomegaly, and splenomegaly [3], [4]. Human and animal brucellosis share many similarities, such as persistence in tissues of the mononuclear phagocyte system, including spleen, liver, lymph nodes, and bone marrow [4]. Therefore, the use of animal models such as mice has been an important tool to better characterize the immune response to *Brucella* infection that leads to long-term bacterial persistence and chronic disease.

There is general agreement that the initial interferon gamma (IFN-γ) mediated Th1 immune response is crucial for the control of *Brucella* infection, since absence of IFN-γ results in decreased control of bacterial growth [5], [6] and IFN-γ-deficient C57BL/6 mice succumb to overwhelming disease [7]. However, the inflammatory response induced by *Brucella* spp. *in vivo* is much milder than that observed with pyogenic infections such as salmonellosis, suggesting the stealth of *Brucella* as a possible reason for the absence of early proinflammatory responses [8], [9]. Recent studies have shown that *Brucella* spp. use both passive and active mechanisms to evade initial innate immune recognition through toll-like receptors (TLRs) [10]. Although avoidance of TLR recognition is a key factor in the lack of initial inflammation during *Brucella* infection, how subsequent interactions of *Brucella* with the host immune system result in chronic disease is poorly understood.

Interleukin-10 (IL-10) is an immunoregulatory cytokine produced by most T cell subsets, B cells, neutrophils, macrophages, and some dendritic cell subsets [11]. It is suggested that by acting on antigen-presenting cells such as macrophages, IL-10 can inhibit the development of Th1 type responses [12]. In the context of infectious diseases, it is believed that the host uses IL-10 to control over-exuberant immune responses to pathogenic microorganisms in order to limit tissue damage [11]. Interestingly, studies using chronic pathogens such as *Leishmania major* [13], human cytomegalovirus [14], or *Mycobacterium tuberculosis* (reviewed in [15]) have demonstrated that the absence of IL-10 leads to a better clearance of these pathogens, with variable degrees of immunopathology. These studies suggest that pathogens have developed mechanisms to take advantage of the host immune-regulation in order to persist for longer periods and establish chronic infection.

Similar to other chronic pathogens, *B. abortus* infection induces IL-10 production [5], [6], [16]. Moreover, IL-10 gene polymorphisms have been associated with increased susceptibility to human brucellosis [17]. However, questions regarding the impact of IL-10 in *B. abortus* persistence and establishment of chronic infection, as well as the cell types responsible for this cytokine production remain to be answered. Therefore, we used IL-10 deficient mice to determine the role of IL-10 in modulating the initial immune response to *Brucella* infection. Furthermore, using cell-specific knock-out mice, we elucidated the immunological mechanisms underlying IL-10 induced immune-regulation during Brucellosis.

## Results

### Lack of IL-10 production during early *B. abortus* infection results in lower bacterial survival and increased pathology *in vivo*

**Figure 1:**
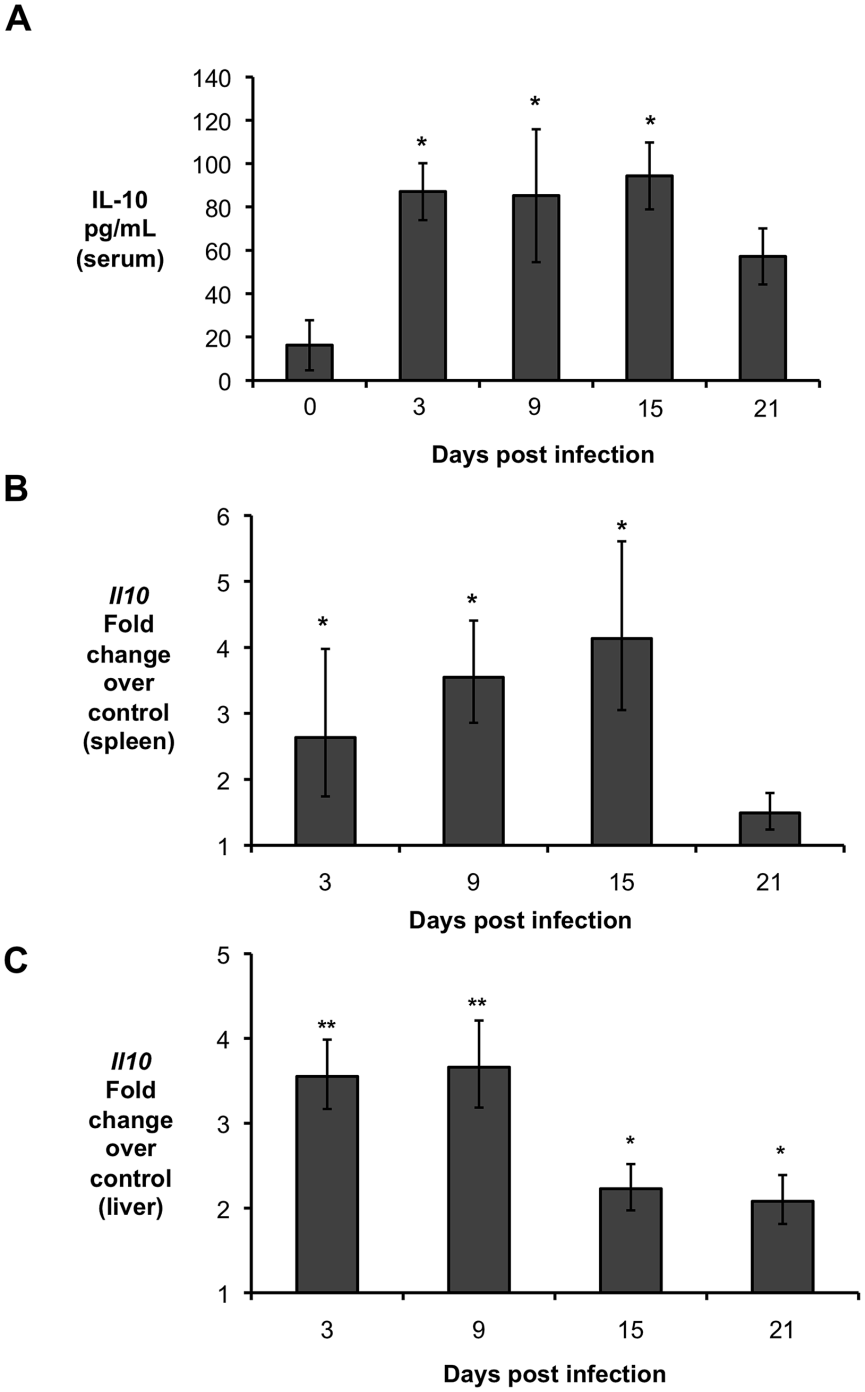
*Brucella abortus* induces IL-10 production by infected organs during early *in vivo* infection. (**A**) IL-10 levels in serum of C57BL/6 infected *with B. abortus* 2308 for 3, 9, 15, and 21 days compared to uninfected mice (inoculated with sterile PBS). (**B, C**) qRT-PCR analysis of IL-10 gene expression in spleen (**B**) and liver (**C**) of C57BL/6 mice infected with *B. abortus* 2308 for 3, 9, 15, and 21 days compared to uninfected controls. n = 5. (*) represents P<0.05 when compared to control. (**) represents P<0.05 when compared to days 15 and day 21 for (**C**) using unpaired t- test statistical analysis.

IL-10 has an important role in controlling the immune response induced by different inflammatory processes [15]. Moreover, *Brucella* infection has been shown to induce IL-10 production by splenocytes *in vitro* [5] and during intravenous *in vivo* infection [6], [16]. To determine the time-course of IL-10 production during *B. abortus* infection, C57BL/6 mice were infected intraperitoneally (IP) with 5×10^5^ CFU of the virulent *B. abortus* strain 2308 and IL-10 production was determined at 3, 9, 15, and 21 days post-infection (d.p.i.). Infected mice exhibited significantly higher levels of IL-10 in the serum (Fig. 1A), which was associated with increased IL-10 transcript levels in the spleen (Fig. 1B) and liver (Fig. 1C) as early as 3 d.p.i. Importantly, significantly increased levels of IL-10 in serum and infected organs was only detected until 15 d.p.i., suggesting a possible regulatory function for this cytokine during acute *Brucella* infection.

**Figure 2:**
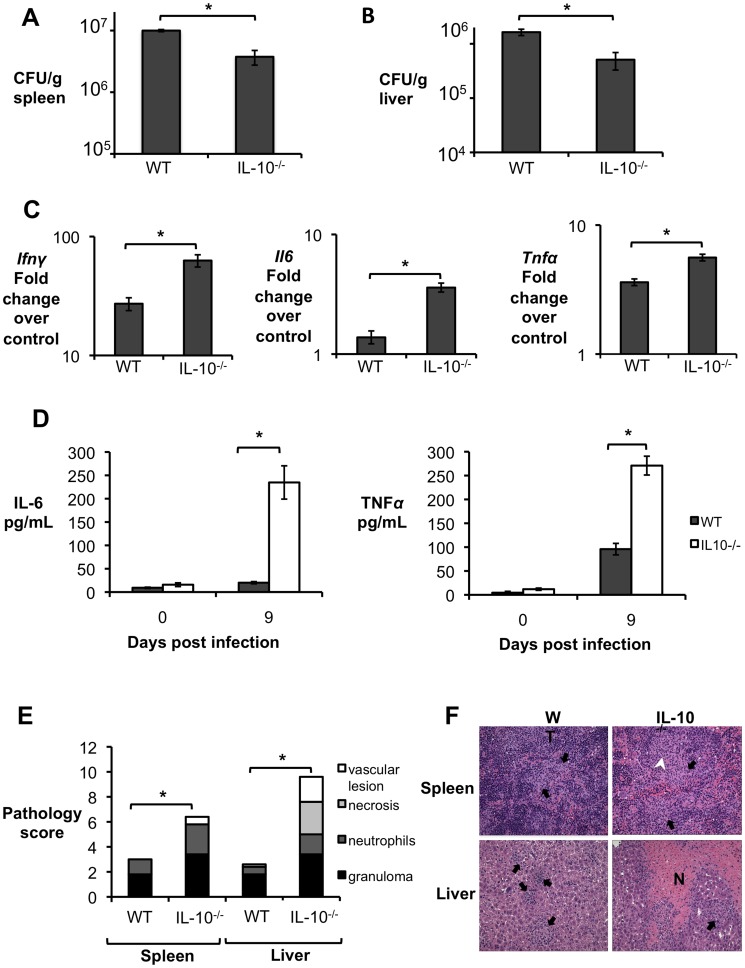
Lack of IL-10 results in lower bacterial survival and increased pathological changes during early *Brucella abortus* infection *in vivo*. (**A,B**) Colonization of spleen (**A**) and liver (**B**) of control or IL-10^−/−^ mice by *B. abortus* at 9 d.p.i. (**C**) qRT-PCR analysis of IFN-γ, IL-6 and TNF-α gene expression in spleens of C57BL/6 and IL-10^−/−^ mice infected with *B. abortus* 2308 for 9 days (similar results for liver). (**D**) IL-6 and TNF-α levels measured by ELISA in serum of C57BL/6 and IL-10^−/−^ mice infected with *B. abortus* 2308 for 9 days. n = 5. Values represent mean ± SEM. *P<0.05 using unpaired t-test statistical analysis.( **E**) Histopathology score of spleen and liver from C57BL/6 and IL-10^−/−^ mice infected with *B. abortus* 2308 for 9 days. (**F**) Representative pictures from (**E**). Black arrow shows microgranulomas, white arrowheads shows neutrophilic infiltrate and upper case N shows areas of coagulative necrosis (×20). n = 5. Values represent individual mice (black circles) and geometric mean (black dash). *P<0.05 using Mann-Whitney statistical analysis.

To further investigate if IL-10 plays a role in modulating the inflammatory response during acute brucellosis, C57BL/6 wild-type and *Il10*-deficient mice (IL-10^−/−^) were infected IP with 5×10^5^ CFU of *B. abortus* 2308 and responses were evaluated at 9 d.p.i. Interestingly, IL-10^−/−^ mice had significantly lower bacterial survival in both the spleen (Fig. 2A) and the liver (Fig. 2B). IL-10^−/−^ mice also exhibited increased induction of pro-inflammatory cytokines such as IFN-γ, interleukin-6 (IL-6) and tumor necrosis factor alpha (TNF-α) in infected organs (Fig. 2C) and in serum from infected mice (Fig. 2D).

The typical tissue response to *Brucella* infection is granulomatous inflammation [4]. However, in the spleen and liver of IL-10^−/−^ mice *B. abortus* infection resulted in development of an acute inflammatory response characterized by vasculitis and thrombosis, necrosis, and influx of neutrophils (Fig. 2E and 2F). Collectively, these data demonstrate a critical role for IL-10 in modulating the initial inflammation and pathology in response to *B. abortus* infection, which in turn benefits the pathogen due to enhanced bacterial survival.

### CD4^+^CD25^+^ T cells are the main IL-10 producers during early *B. abortus* infection *in vivo*

**Figure 3:**
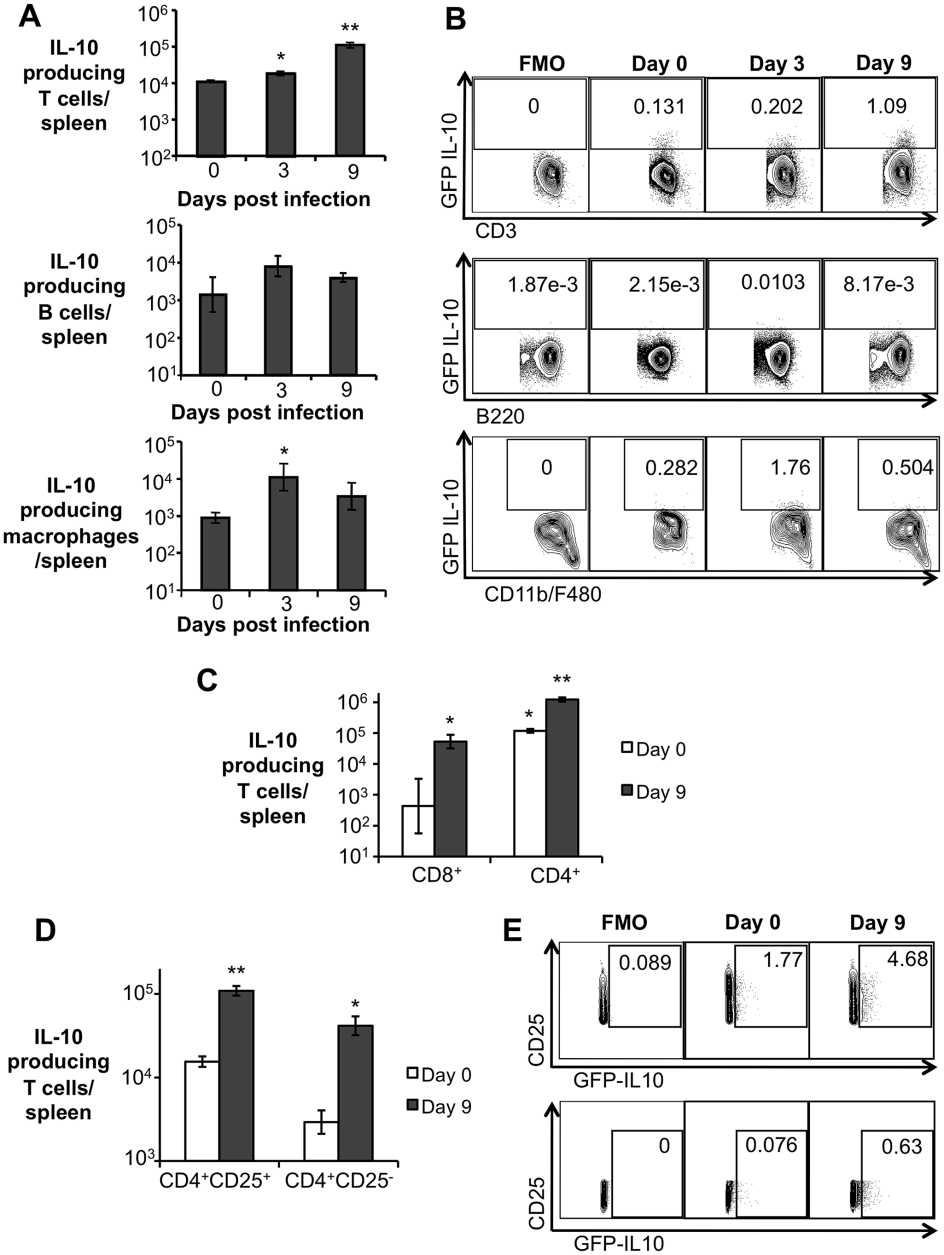
CD4^+^CD25^+^ T cells are the main producers of IL-10 during early *Brucella abortus in vivo* infection. (**A**) Flow cytometry measurement of IL-10 expression in splenic T cells, B cells, and macrophages from C57BL/6 IL-10 GFP-reporter mice infected with *B. abortus* 2308 for 3 and 9 days. (**B**) Representative data plot of IL-10 expression in splenic T cells (CD3), B cells (B220), and macrophages (CD11b^+^F4/80^+^ cells) from C57BL/6 IL-10 GFP-reporter mice. (**C**) Flow cytometry measurement of IL-10 expression in splenic CD8^+^ T cells and CD4^+^ T cells from C57BL/6 IL-10 GFP-reporter mice infected with *B. abortus* 2308 for 9 days. (**D**) Flow cytometry measurement of IL-10 expression in splenic CD4^+^CD25^+^ T cells and CD4^+^CD25^−^ T cells from C57BL/6 IL-10 GFP-reporter mice infected with *B. abortus* 2308 for 9 days. (**E**) Representative data plot of IL-10 expression in splenic CD4^+^CD25^+^ (upper panel) and CD4^+^CD25^−^ (lower panel) T cells from C57BL/6 IL-10 GFP-reporter mice. Values represent mean ± SEM. n = 4. (*) represents P<0.05 relative to uninfected control (day 0), (**) represent P<0.05 relative to day 3 infection for (**A**), relative to CD8^+^ day 9 infected for (**C**) and relative to CD4^+^CD25^−^ for (**D**) using unpaired t-test statistical analysis. FMO = fluorescence minus one.

IL-10 can be produced by different T cell subsets, as well as by B cells, neutrophils, macrophages, and some DC subsets [18]. To determine the cell types responsible for IL-10 production during early *B. abortus* infection, *Il10*-GFP reporter mice [19] were infected IP with 5×10^5^ CFU of *B. abortus* and IL-10 producing cells were identified at 3 and 9 d.p.i. by flow cytometry. A significant increase in the number of IL-10 producing T cells was observed in infected mice at 3 and 9 days post infection, whereas macrophages presented increased production of IL-10 only at 3 d.p.i. Moreover, the number of IL-10 producing B cells, neutrophils, and dendritic cells did not change significantly when compared to uninfected mice (Fig. 3A and 3B, and data not shown). Importantly, even though a significant increase in the number of IL-10 producing CD8^+^ T cells was observed (Fig. 3C), a tenfold higher number of IL-10 producing T cells was observed in the CD4^+^ T cell population (Fig. 3C). No IL-10 production by γδ T cells was observed (data not shown).

Various IL-10 producing CD4^+^ T cells have been described, including the CD4^+^CD25^+^ subset [20]. Interestingly, an expansion of the CD4^+^CD25^+^ T cell population was observed in the spleen of *B. abortus* infected mice at 9 d.p.i. (Fig. S1). Moreover, a 5-fold greater number of IL-10 producing CD4^+^CD25^+^ T cells was detected at 9 d.p.i., compared to IL-10 producing CD4^+^CD25^−^ T cells (Fig. 3D and 3E), suggesting that the CD4^+^CD25^+^ T cell subset is the major population responsible for IL-10 production during acute brucellosis in the mouse.

### IL-10 production by T cells is required for *B. abortus* persistence and for pathology regulation *in vivo*

**Figure 4:**
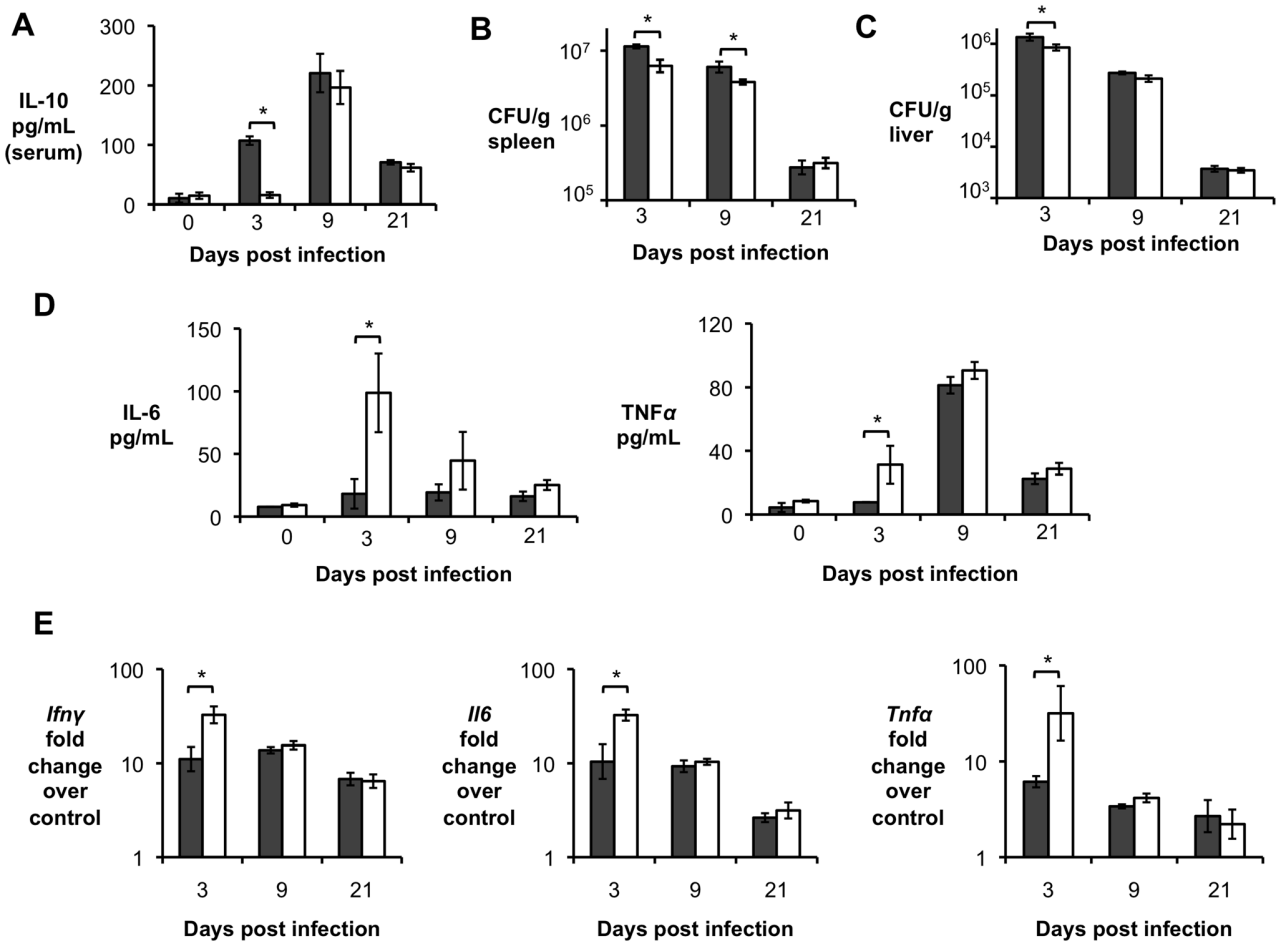
IL-10 production by macrophages and/or neutrophils is not required for *Brucella abortus* long-term persistence *in vivo*. (**A**) ELISA essay for IL-10 production in serum from littermate control mice (grey bars) compared with IL10flox/LysMCre mice (white bars) at 3, 9 and 21 d.p.i. (**B, C**) *B. abortus* 2308 CFU counts in spleen (**B**) and liver (**C**) from littermate control mice (grey bars) compared with IL10flox/LysMCre mice (white bars) at 3, 9 and 21 d.p.i.). (**D**) ELISA essay for IL-6 and TNF-α production in serum from littermate control mice (grey bars) compared with IL10flox/LysMCre mice (white bars) at 3, 9 and 21 d.p.i. (**E**) RT-PCR analysis of pro-inflammatory cytokines in spleens from littermate control mice (grey bars) compared with IL10flox/LysMCre mice (white bars) at 3, 9 and 21 d.p.i.

Our previous data suggested that T cells and possibly macrophages would be the main cell types producing IL-10 during early *Brucella* infection. Therefore, to further investigate the importance of macrophage derived IL-10 during *Brucella* infection, we generated *Il-10* ^flox/flox^ *LysMCre* ^+/−^ (IL-10flox/LysMCre) mice, which have macrophages and neutrophils that are unable to produce IL-10. IL-10flox/LysMCre mice and littermate *Il-10* ^flox/flox^ *LysMCre* ^−/−^ controls were infected IP with 5×10^5^ CFU of *B. abortus* 2308, and at 3, 9 and 21 d.p.i. disease progression was evaluated. Interestingly, IL-10flox/LysMCre infected mice exhibited decreased levels of IL-10 in the serum (Fig. 4A) when compared to control animals only at 3 d.p.i., Moreover, IL-10flox/LysMCre mice exhibited increased ability to control *B. abortus* infection in both spleen (Fig. 4B) and liver (Fig. 4C), only at initial stages of infection. This increased host resistance was accompanied by significantly higher levels of the pro-inflammatory cytokines IL-6, TNF-α and IFN-γ in serum (Fig. 4D), spleen (Fig. 4E) and liver (data not shown) as well as increased histopathological lesions in infected organs (Fig. S2) of IL-10flox/LysMCre mice when compared to littermate controls (Fig. 4D, 4E and data not shown). Collectively, this data suggests that macrophage derived IL-10 plays a limited role in development of the chronic disease caused by *B. abortus* and raises the possibility that T-cells could, indeed, be the main cell type responsible for IL-10 production during early Brucellosis.

Therefore, to further investigate the importance of T-cell derived IL-10 during *Brucella* infection, we used *Il-10* ^flox/flox^ *Cd4Cre^+^* ^/−^(IL-10flox/CD4Cre) mice, which have T cells that are unable to produce IL-10 [21]. IL-10flox/CD4Cre and littermate *Il-10* ^flox/flox^ *Cd4Cre^−^* ^/−^ control mice were infected IP with 5×10^5^ CFU of *B. abortus* 2308, and at 3, 9 ,21 and 42 d.p.i., disease progression was evaluated. Remarkably, IL-10flox/CD4Cre infected mice exhibited lower levels of IL-10 in serum (Fig. S3A), and spleen (Fig. S3B) when compared to control animals at 9 d.p.i., providing compelling support for the hypothesis that T-cells are a major source of IL-10 production during early *B. abortus* infection.

**Figure 5:**
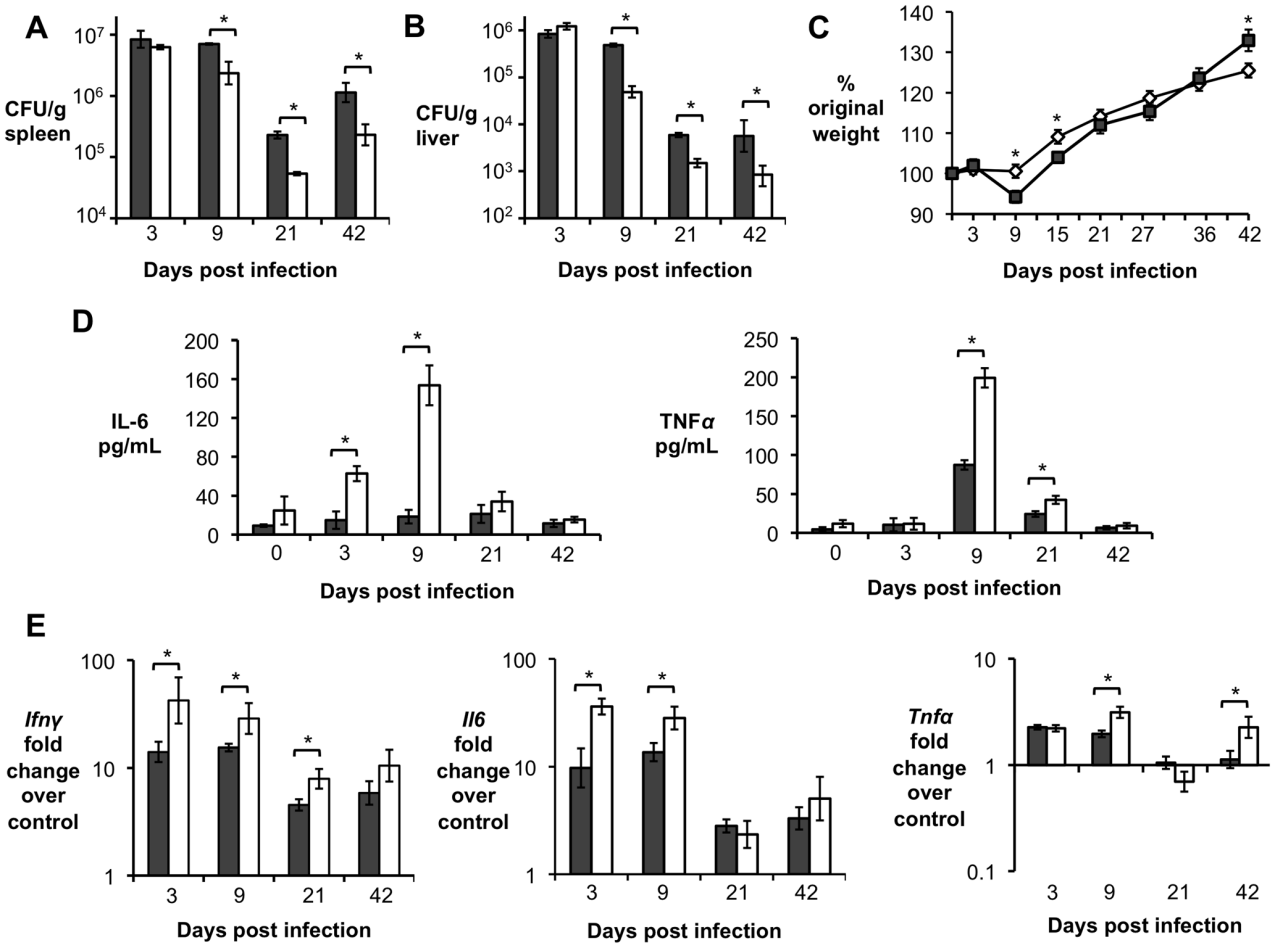
IL-10 production by T cells is required for *Brucella abortus* persistence and for control of pathology *in vivo*. (**A, B**) *B. abortus* 2308 CFU counts in spleen (**A**) and liver (**B**) from littermate control (grey bars) compared with IL10flox/CD4Cre mice (white bars) at 3, 9, 21 and 42 d.p.i. (**C**) Measurement of mouse weight over time in grams of littermate control mice (grey dots) compared with IL10flox/CD4Cre mice (white dots). (**D**) ELISA essay for IL-6 and TNF-α production in serum from littermate control mice (grey bars) compared with IL10flox/CD4Cre mice (white bars) at 3, 9, 21 and 42 d.p.i. (**E**) qRT-PCR analysis of pro-inflammatory cytokines genes (*Ifnγ*, *Il6* and *Tnfα*) in spleen from littermate control mice (grey bars) compared with IL10flox/CD4Cre mice (white bars) at 3, 9, 21 and 42 d.p.i. n = 5. Values represent mean ± SEM. (*) represents P<0.05 using unpaired t-test statistical analysis.

Previous results using IL-10^−/−^ and IL-10flox/LysMCre mice (Fig. 2 and 4) suggested that IL-10 was important for initial *B. abortus* persistence in the host. Therefore, we investigated whether the lack of T cell-derived IL-10 would affect bacterial persistence. Indeed, IL-10flox/CD4Cre mice exhibited significantly improved control of *B. abortus* infection in the spleen and liver at 9, 21 and 42 d.p.i. (Fig. 5A and 5B).

Controlling *B. abortus* replication could be beneficial to the host, since the ability to survive for longer periods is a key mechanism for chronic pathogens to thrive. However, loss of IL-10 driven immune modulation has been shown to cause severe and sometimes lethal inflammatory responses in different infectious disease models [15]. To determine the disease progression, weight changes in IL-10flox/CD4Cre and control mice were followed at 3, 9, 15, 21, 28, 35 and 42 d.p.i. (Fig. 5C). To ensure that any detectable change in weight was the result of *B. abortus* infection, uninfected IL-10flox/CD4Cre and littermate control mice were also used (data not shown). IL-10flox/CD4Ce mice exhibited significantly decreased weight gain during early infection and, by 21 days post-infection, they started to behave like control animals. IL-10flox/CD4Cre uninfected mice did not exhibit any slower weight gain and behaved like uninfected control mice (data not shown). Moreover, increased levels of the pro-inflammatory cytokines IFN-γ, IL-6, and TNF-α were observed in serum (Fig. 5D) spleen (Fig. 5E) and liver (Fig. S3C) of IL-10flox/CD4Cre at 3, 9 days and, to a lesser extent, at 21 d.p.i.

**Figure 6:**
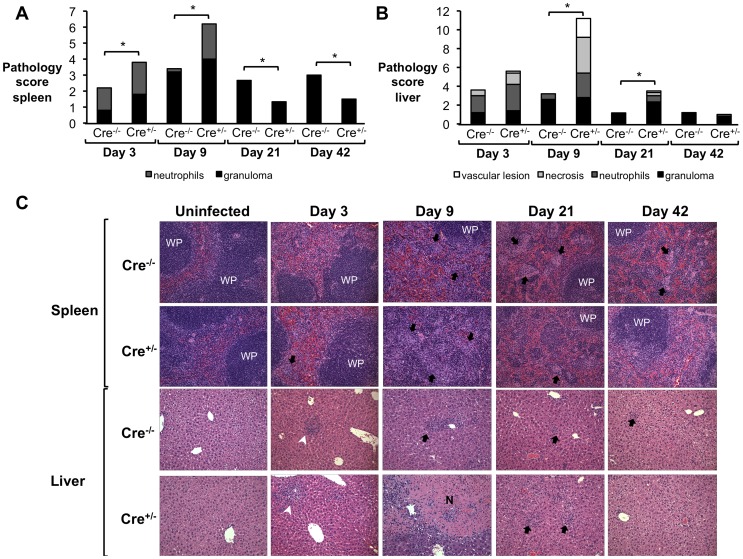
IL-10 production by T cells is required for control of *Brucella abortus* induced pathology *in vivo*. (**A, B**) Histopathology score of spleen (**A**) and liver (**B**) from littermate mice (Cre^−/−^) compared with IL10flox/CD4Cre mice (Cre^+/−^) at 3, 9, 21 and 42 d.p.i. (**C**) Representative histopathology figures from (**A,B**) - Black arrows indicate microgranulomas, white arrowheads show neutrophilic infiltrate, and white upper case WP indicates white pulp (×20). n = 5. (*) represents P<0.05 using Mann-Whitney statistical analysis.

To determine if the significantly reduced weight gain and higher induction of pro-inflammatory cytokines were associated with a detrimental inflammatory response, spleen and liver sections from IL-10flox/CD4Cre and control mice were blindly evaluated by veterinary pathologists (MNX and TMS). Interestingly, IL-10flox/CD4Cre mice exhibited increased pathology, characterized by marked influx of neutrophils and histiocytes in the spleen (Fig. 6A and 6C), as well as tissue necrosis and multifocal neutrophilic vasculitis and thrombosis in the liver (Fig. 6B and 6C) at 3 and 9 d.p.i., suggesting an acute inflammatory response. Importantly, a hallmark of *B. abortus* infection is a mild initial pro-inflammatory response, which leads to a chronic infection characterized by formation of granulomas in infected organs [4], [22]. However, by 21 d.p.i., IL-10flox/CD4Cre mice exhibited decreased granuloma formation in the spleen (Fig. 6A and 6C) when compared to littermate control mice. Taken together, these data demonstrate that T cell-derived IL-10 production during early *B. abortus* infection is crucial for the development of the chronic disease and morbidity caused by *B. abortus*, and limits the production of the pro-inflammatory response necessary to control the infection. However, the cell types affected by the IL-10 production during *Brucella* infection remained unclear.

### Lack of IL-10 results in lower *B. abortus* survival in infected macrophages due to bacterial inability to escape late endosome

**Figure 7:**
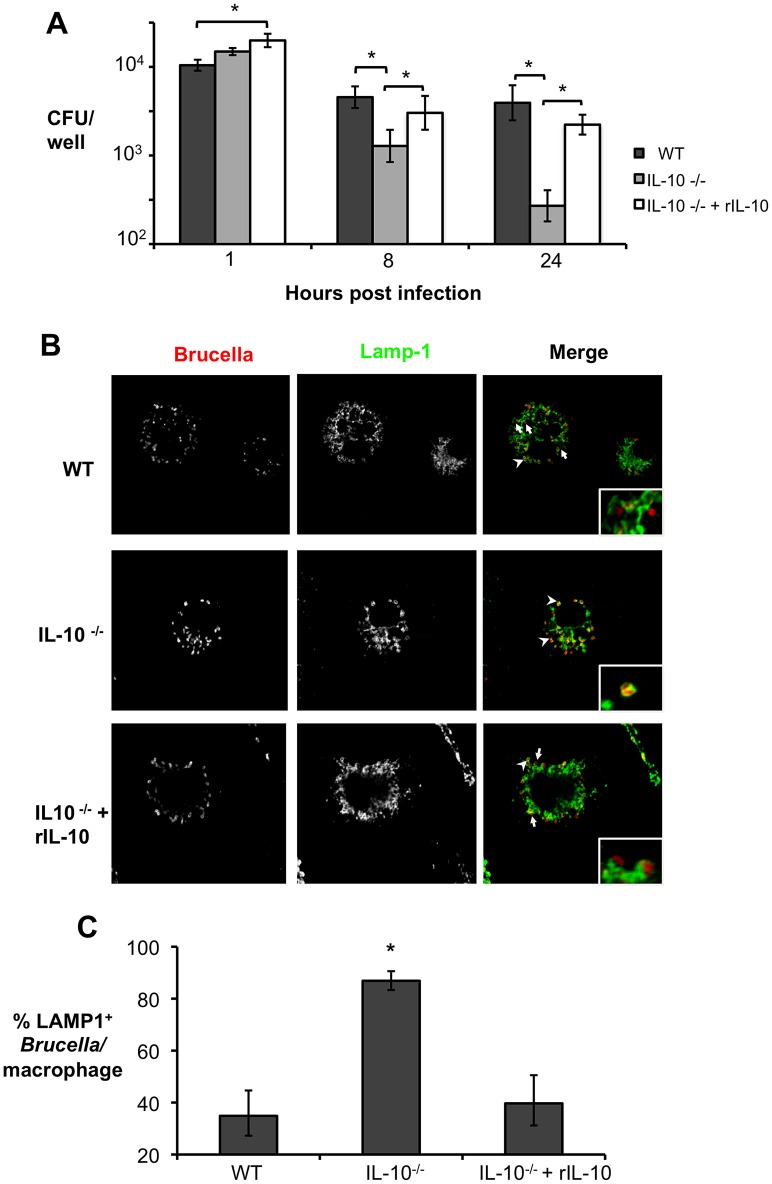
Lack of endogenous IL-10 results in lower *Brucella abortus* survival inside macrophages due to bacterial inability to escape the late endosome. (**A**) Survival of *B. abortus* over time in BMDM from C57BL/6 WT control mice and IL-10 deficient mice with (IL-10^−/−^+rIL-10) or without (IL-10^−/−^) rIL-10 added to the culture. (**B**) Confocal microscopy of BMDM from C57BL/6 WT control mice and IL-10 deficient mice with (IL-10^−/−^+rIL-10) or without (IL-10^−/−^) added rIL-10, infected with mCherry-expressing *B. abortus* 2308 for 24 h. Colocalization of the bacteria (red) with the late endosomal marker LAMP-1 (green) is shown by arrowheads, while arrows indicate bacteria that were able to escape the endosomal compartment. Intracellular survival of *B. abortus* in each treatment group is shown in Panel (**A**). (**C**) Quantification of % of colocalization of mCherry-expressing *B. abortus* and LAMP1 in infected macrophages shown in (**B**). Representative results of two independent experiments are shown. n = 4. Values represent mean ± SEM. *P<0.05 using unpaired t-test statistical analysis.

Since *Brucella* spp. are known to invade and survive inside phagocytic cells such as macrophages [23], we hypothesized that macrophages could be the cell type affected by IL-10 during *Brucella* infection. To determine the effect of IL-10 on *B. abortus* survival during macrophage infection *in vitro*, bone marrow derived macrophages (BMDM) from C57BL/6 and IL-10^−/−^ mice were infected with *B. abortus* 2308 (MOI = 100) and the bacterial survival was measured at 1, 8, and 24 h post-infection (h.p.i.) (Fig. 7A). *B. abortus* infected wild-type BMDM produced significant amounts of IL-10 (Fig. S4A). *B. abortus* exhibited a significantly decreased ability to survive inside BMDM from IL-10^−/−^ mice at 8 and 24 h.p.i. when compared to BMDM from wild-type mice. Importantly, *B. abortus* infected IL-10^−/−^ BMDM did not exhibit increased cell death, determined by LDH assay (data not shown). Moreover, to ensure that the observed effect resulted from an absence of IL-10 production, recombinant IL-10 (rIL-10) was added to the IL-10^−/−^ BMDM media during infection (Fig. 7A). As expected, the addition of rIL-10 restored *B. abortus* survival inside macrophages. Results similar to those described above (Fig. 7A) were also observed in IFNγ-activated BMDM (data not shown).

The ability of *Brucella* spp. to persist and replicate within macrophages involves a temporary fusion of *Brucella*-containing vacuole with the late endosome/lysosome during the initial hours post-infection, and subsequent exclusion of the endosomal/lysosomal proteins from the *Brucella*-containing vacuole [24]. To determine if *B. abortus* inability to persist inside macrophages was due to changes in the pathogen's intracellular trafficking, we infected wild-type and IL-10^−/−^ BMDM with *B. abortus* expressing mCherry (MOI = 100) and bacterial co-localization with the late endosome marker LAMP1 was determined at 24 h.p.i. by confocal microscopy. Interestingly, while the majority of *B. abortus* was found to lack LAMP1 in wild type BMDM, the opposite was found in BMDM derived from IL-10^−/−^ mice, in which over 80% of bacteria were co-localized with LAMP1 (Fig. 7B and 7C). This phenotype was IL-10-dependent, since addition of rIL-10 to the IL-10^−/−^ BMDM restored the ability of the pathogen to escape the late endosome (Fig. 7B and 7C). Results similar to those described (Fig. 7B and Fig. 7C) were also observed in IFNγ activated BMDM (data not shown).

### Lack of IL-10 results in higher levels of NF-κB activation and pro-inflammatory cytokine production in *B. abortus* infected macrophages

**Figure 8:**
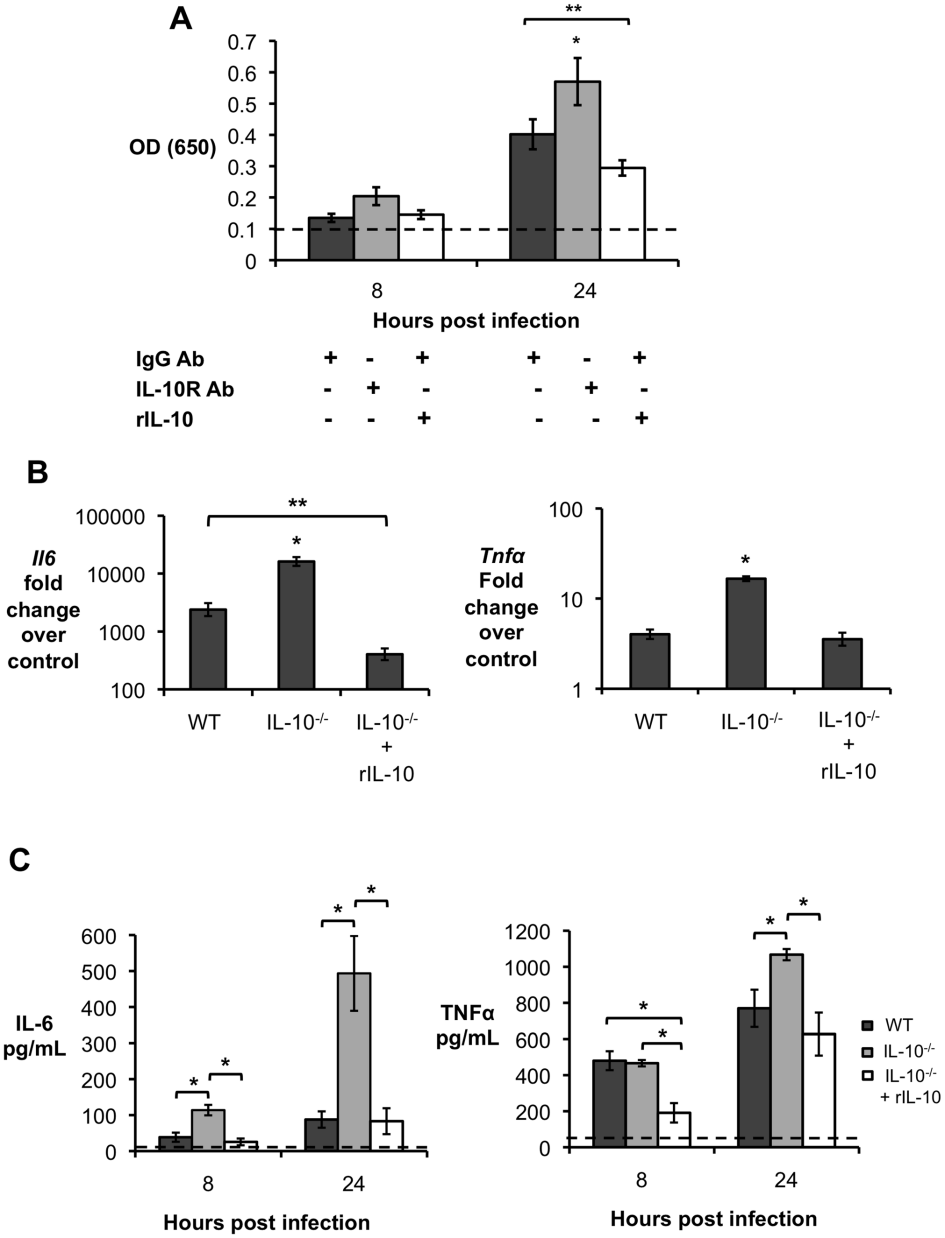
Lack of endogenous IL-10 results in higher NF-κB activation and production of pro-inflammatory cytokines by macrophages infected with *B. abortus*. (**A**) NF-κB activation measured in RAW-Blue macrophages infected with *B. abortus* 2308 for 8 h and 24 h in the presence of IL-10 receptor blocking antibody (IL-10R Ab), isotype control (IgG Ab) or exogenous IL-10 (rIL-10). (**B**) qRT-PCR analysis of IL-6, and TNF-α expression by BMDM from wild type mice (C57BL/6) and IL-10 deficient mice (IL-10^−/−^) in the presence or absence of recombinant IL-10. (**C**) ELISA essay for IL-6 and TNF-α production in supernatant from wild type mice (C57BL/6) and IL-10 deficient mice (IL-10^−/−^) in the presence or absence of recombinant IL-10. Results shown are representative of two independent experiments. n = 5. Values represent mean ± SEM. *P<0.05 using unpaired t-test statistical analysis.

It has been demonstrated that IL-10 can inhibit a protective immune response, possibly by blocking NF-κB activation [25] and downstream production of pro-inflammatory cytokines by antigen-presenting cells such as macrophages [15], [26]. To investigate if IL-10 production would have an effect on NF-κB activation in *Brucella* infected macrophages, we used an NF-κB reporter RAW murine macrophage cell line (RAW-Blue cells). RAW-Blue cells were infected with *B. abortus* 2308 (MOI = 100) in the presence of IL-10 receptor blocking antibody (IL-10R Ab) or rIL-10, and NF-κB activation was measured at 8 and 24 h.p.i. *Brucella* infected RAW-Blue macrophages produced significant amounts of IL-10 (Fig. S4B). Blockage of IL-10R resulted in decreased *B. abortus* intracellular survival inside RAW-Blue macrophages when compared to untreated controls at 24 h.p.i., as previously observed in IL-10^−/−^ BMDM (data not shown). *B. abortus* infection did not result in significant NF-κB activation by any of the treatment groups at 8 h.p.i. (Fig. 8A). The use of IL-10R Ab to block the response of *B. abortus* infected RAW-Blue macrophages to IL-10 resulted in a significant increase in NF-κB activation at 24 h.p.i. when compared to untreated infected macrophages. Conversely, addition of exogenous rIL-10 resulted in a significant inhibition of NF-κB activation in *B. abortus* infected cells (Fig. 8A). The results above described were also observed in IFNγ activated RAW blue cells (data not shown).

To confirm that IL-10 affects pro-inflammatory cytokine production by infected macrophages, bone marrow-derived macrophages (BMDM) from C57BL/6 and IL-10^−/−^ mice were infected with *B. abortus* 2308 (MOI = 100) and cytokine expression was measured at 24 h.p.i. by ELISA and quantitative real-time PCR. Significantly, an absence of IL-10 resulted in higher expression levels of the pro-inflammatory cytokines IL-6 and TNFα by infected macrophages (Fig. 8B and 8C). Moreover, the phenotype observed was shown to be IL-10 dependent, since the addition of rIL-10 to IL-10^−/−^ infected BMDM restored IL-6 and TNF-α expression to wild-type levels. Results similar to those described in Fig. 8B and Fig. 8C were also observed in IFN-γ activated BMDM (data not shown). Our results demonstrate that IL-10 production during *B. abortus* infection *in vitro* affects macrophage function by modulating NF-κB activation and the production of pro-inflammatory cytokines by infected cells.

### Inability of macrophages to respond to IL-10 results in severe acute pathology and decreased *B. abortus* survival *in vivo*

**Figure 9:**
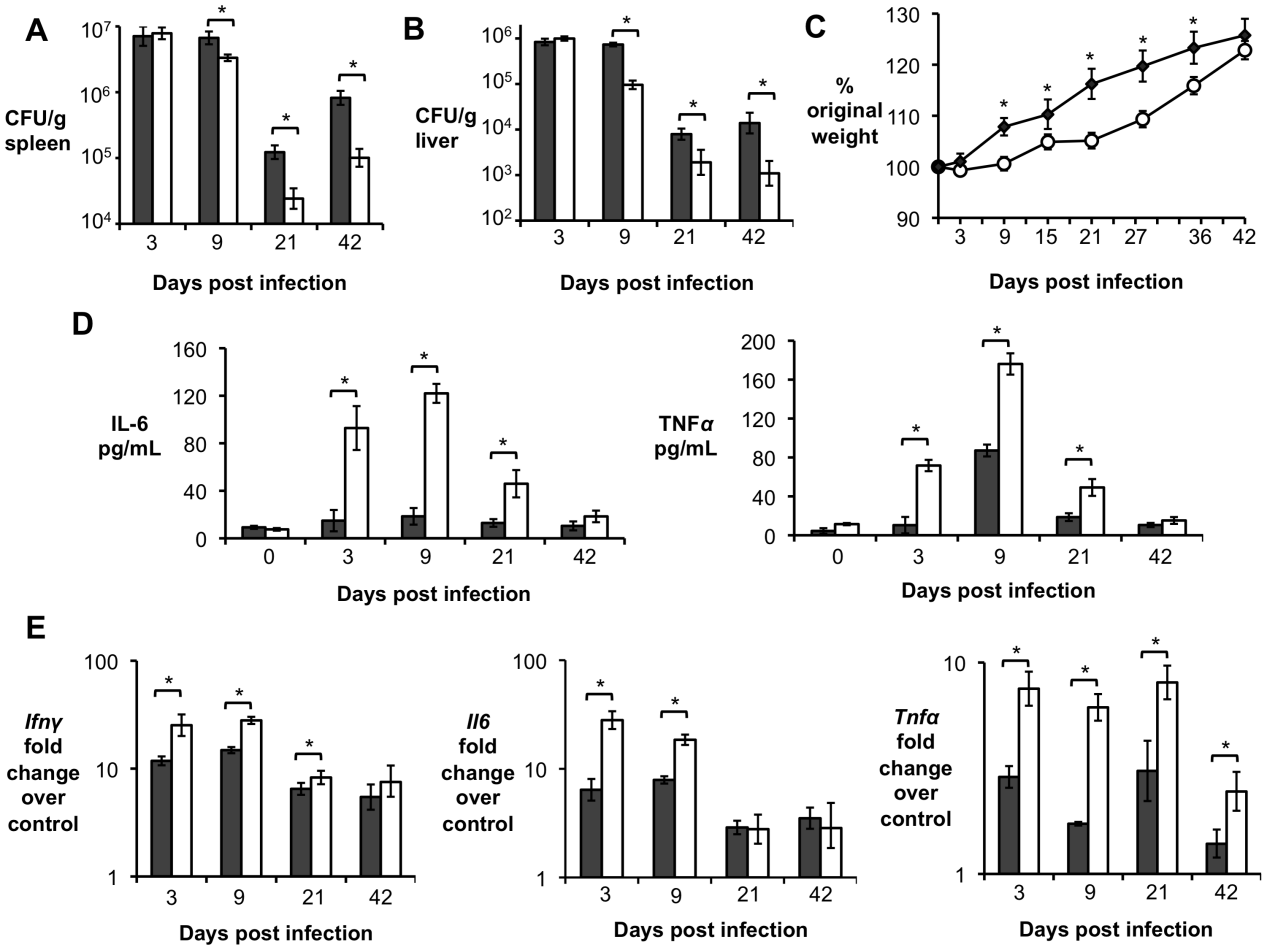
Inability of macrophages to respond to IL-10 results in decreased persistence of *B. abortus in vivo*. (**A,B**) *B. abortus* 2308 CFU counts in spleen (**A**) and liver (**B**) from littermate control mice (grey bars) compared with IL10Rflox/LysMCre (white bars) at 3, 9, 21 and 42 d.p.i. (**C**) Measurement of mouse weight over time in grams of littermate control mice (grey losangle) compared with IL10Rflox/LysMCre (white dots). (**D**) ELISA essay for IL-6 and TNF-α production in serum from littermate control mice (grey bars) compared with IL10Rflox/LysMCre mice (white bars) at 3, 9, 21 and 42 d.p.i. (**E**) qRT-PCR analysis of pro-inflammatory cytokines genes (*Ifnγ*, *Il6* and *Tnfα*) in spleen from littermate control mice (grey bars) compared with IL10Rflox/LysMCre mice (white bars) at 3, 9, 21 and 42 d.p.i. n = 5. Values represent mean ± SEM. (*) represents P<0.05 using unpaired t-test statistical analysis.

To further investigate the possibility that macrophages are the cell type responding to the IL-10 produced during *B. abortus* infection, we used *Il-10R* ^flox/flox^ *LysMCre* ^+/−^ (IL-10Rflox/LysMCre) mice, which are unable to express the IL-10 receptor 1 chain (IL-10R1) specifically in monocytes/macrophages and/or neutrophils [27]. IL-10Rflox/LysMCre and *Il-10R* ^flox/flox^ *LysMCre* ^−/−^ control mice were infected intraperitoneally with 5×10^5^ CFU of *B. abortus* 2308 for 3, 9, 21 and 42 days and bacterial survival in infected organs was evaluated. Our results from *in vitro* infection suggested that IL-10 affects the ability of *B. abortus* to survive inside macrophages. Remarkably, IL-10Rflox/LysMCre mice showed lower CFU counts in both spleen (Fig. 9A) and liver (Fig. 9B) at 9, 21 and 42 d.p.i. when compared to littermate control mice. This data provided strong support for the idea that macrophage responsiveness to IL-10 is necessary for optimal initial *B. abortus* colonization of the host as well as long-term persistence.

The results shown above (Fig. 5) demonstrated that a lack of IL-10 production by T cells during *B. abortus in vivo* infection resulted in increased pro-inflammatory responses and evident clinical signs of disease in mice. Moreover, our *in vitro* results suggested that blockage of IL-10R played a role in the control of NF-κB activation and pro-inflammatory cytokine production by *B. abortus*-infected macrophages. Therefore, we sought to determine the effect of macrophage responsiveness to IL-10 in the early host response to *B. abortus* infection. *B. abortus*-infected IL-10R/LysMCre mice exhibited decreased weight gain at 9, 15, and 21 d.p.i. when compared to wild-type infected mice (Fig. 9C). Furthermore, levels of IFN-γ, IL-6 and TNF-α were significantly increased in serum (Fig. 9D) spleens (Fig. 9E) and livers (Fig. S5) of IL-10R/LysMCre at 3, 9 d.p.i. and, to a lesser extent, at 21 d.p.i.

**Figure 10:**
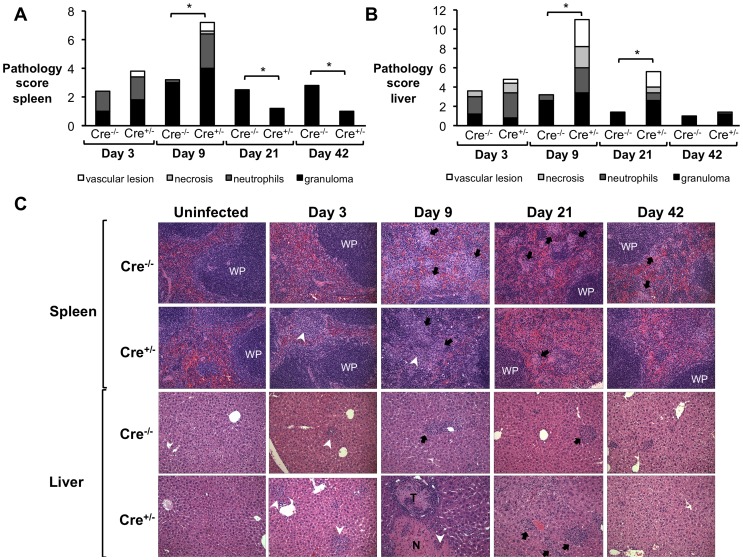
Inability of macrophages to respond to IL-10 results in severe acute *B. abortus* induced pathology *in vivo*. (**A, B**) Histopathology score of spleen (**A**) and liver (**B**) from littermate mice (Cre^−/−^) compared with IL10Rflox/LysMCre mice (Cre^+/−^) at 3, 9, 21 and 42 d.p.i. (**C**) Representative histopathology figures from (**A,B**) - Black arrows indicate microgranulomas, white arrowheads show neutrophilic infiltrate and white upper case WP indicates white pulp (×20). n = 5. (*) represents P<0.05 using Mann-Whitney statistical analysis.

To determine if macrophage non-responsiveness to IL-10 would result in detrimental pathologic changes, spleen and liver sections from infected IL-10Rflox/LysMCre and control mice were blindly evaluated by veterinary pathologists (MNX and TMS). As expected, IL-10Rflox/LysMCre showed severe acute lesions characterized by influx of neutrophils and histiocytes, as well as tissue necrosis and multifocal neutrophilic vasculitis and thrombosis in the spleen at 9 d.p.i. (Fig. 10A and 10C) and in the liver (Fig. 10B and 10C) at 9 and 21 d.p.i. However, at 21 and 42 days post-infection, IL-10Rflox/LysMCre mice exhibited decreased granuloma formation in spleen (Fig. 10A and 10C) when compared to littermate control mice, suggesting that macrophage responsiveness to IL-10 is important for development of chronic pathological lesions in spleens of infected animals. These data provide the direct support for the idea that induction of IL-10 during *B. abortus in vivo* infection plays a key role in modulation of macrophage function, which, in turn, provides the ideal initial immunological environment for bacterial colonization and development of chronic infection.

## Discussion

The balance between pro-inflammatory and anti-inflammatory cytokine production appears to be crucial for the ability of the host to eradicate an infection, as well as for the clinical presentation and/or pathology resulting from the infection. This balance appears to shift in the case of persistent pathogens such as *Brucella* spp., which are able to evade TLR signaling during the early stages of infection, thereby preventing development of an immune response that is appropriate to clear the infection [28]. It is known that during the acute phase of *B. abortus* infection in mice, neutralization of IL-10 reduces bacterial colonization [5]. Here, we provide support to the idea that during this phase of *B. abortus* infection, early production of IL-10 by T cells is key to promoting persistent intracellular infection.

In a number of infectious disease models, several cell types, including T cell subsets, B cells, neutrophils, macrophages and some DC subsets have been shown to be able to produce IL-10 [18]. Even though *B. abortus* infected macrophages are capable of producing IL-10 *in vitro*, only B cells have been implicated as a potential source of IL-10 during *in vivo* infection [29]. In this study, however, we have demonstrated that macrophages play a limited role in IL10 production during early acute *B. abortus* infection. Additionally, we identified T cells, more specifically CD4^+^CD25^+^ T cells, as the major source of this cytokine during acute brucellosis. Indeed, Svetić and collaborators [1] have suggested a possible role for CD4+ T cells in IL-10 production during acute murine Brucellosis. Interestingly, previous studies have demonstrated elevated numbers of CD4+CD25+ T cells in PBMCs from human patients with acute brucellosis [30] as well as in draining lymph nodes from *B. melitensis* infected sheep [28]. Moreover, Pasquali and collaborators demonstrated that depletion of CD4^+^CD25^+^ T cells resulted in increased control of *B. abortus* infection due to elevated activation of effector T cells and higher production of pro-inflammatory cytokines such as IFN-γ by infected mice [31]. Most likely, the effects seen in this latter study were an indirect effect of the decreased IL-10 production by T cells, resulting in elevated macrophage activation in CD25-depleted mice upon *B. abortus* infection. Taken together, these results point to CD4^+^CD25^+^ T cells as important players in modulating the early immune response to *B. abortus in vivo*.

There is a general agreement that macrophages represent a critical niche for *Brucella* persistence in the host [4]. Importantly, they have been described as one of the cell types responding to IL-10 production in other infection models [11], [27]. Here we have demonstrated that macrophages are a main cell type responding to the immunomodulatory functions of IL-10 during both *in vitro* and *in vivo Brucella* infection. Moreover, IL-10 signaling had a significant impact on the ability of *B. abortus*-infected macrophages to produce pro-inflammatory cytokines and to permit intracellular growth of *B. abortus*. Interestingly, O'Leary and collaborators have demonstrated that IL-10 production by immune cells can affect the ability of *M. tuberculosis* to escape the LAMP1^+^ late endosomal compartment and to establish infection in human macrophages *in vitro* [32]. In agreement with this previous study, we have demonstrated that the capacity of macrophages to respond to IL-10 impacts intracellular survival of *Brucella* by decreasing the pathogen's ability to escape the LAMP1^+^ late endosome, a prerequisite for replication in an endoplasmic reticulum-associated compartment.

It is important to note that the LysM promoter used to drive Cre expression in IL10Rflox mice is expressed in both neutrophils and macrophages [27]. Although our understanding of the role of neutrophils during brucellosis is still evolving [8], [33], we and others have described recruitment of these cells to both spleens and livers of *B. abortus*-infected mice during the acute infection phase ([33], Fig. 6 and Fig. 9). Moreover, the neutrophil recruitment was more evident in the absence of IL-10 (Fig. 2, Fig. 6 and Fig. 9). Therefore, although *Brucella* resistance to neutrophil killing has been well described [34], it is possible that the increased cytokine expression and pathology observed in IL10Rflox/LysMCre mice could also be due in part to a failure of neutrophils recruited to the site of infection to respond to IL-10.

It should be pointed out that, although IL-10 contributes to persistence of *B. abortus in vivo*, abrogation of IL-10 production (Fig. 2) or neutralization of IL-10 in vivo [5] did not result in eradication of *B. abortus* from tissues, contrary to what has been shown for *Leishmania* [13]. Therefore, factors in addition to IL-10 production must also contribute to chronic persistence of *B. abortus*. Although TGF-β has been shown to be produced by B cells and macrophages in BALB/c mice [29], consistent with this report, we did not observe any increase in circulating TGF-β1, TGF-β2, or TGF-β3 at 21 or 42 days post infection of C57BL/6 mice (data not shown). However, these results do not rule out a role for local activation of TGF-β in promoting chronic infection. An important factor in persistence of *Brucella* is the transient nature of IFNγ production in infected mice, which subsides by 21 d post infection in mice [35], therefore our observed lack of a role of IL-10 later in infection could suggest that its role is to antagonize the activity of IFNγ at earlier stages of infection. Finally, the possibility should be considered that during chronic infection, *B. abortus* may reside in a cell type that has inherently low microbicidal activity, as has been found for *M. tuberculosis* [36] and *B. melitensis* [37].

At first glance, our data suggest that inhibition of IL-10 signaling would be beneficial to the host, since IL-10^−/−^, IL-10flox/CD4Cre and IL-10Rflox/LysMcre showed increased ability to control *B. abortus* infection at both acute and chronic stages of infection. Moreover, both IL-10flox/CD4Cre and IL-10Rflox/LysMcre exhibited reduced formation of granulomas, a potential niche for *B. abortus* persistence [4], during the chronic stage of infection. However, in spite of the increased bacterial clearance, we demonstrated that lack of IL-10 during *Brucella* infection could potentially be detrimental to the host, since *B. abortus* infected IL-10 deficient mice presented evident signs of acute disease, characterized by changes in weight gain and marked histopathological lesions in both spleen and liver. Indeed, studies on other chronic pathogens such as *Leishmania major* [38], human cytomegalovirus [14], and *M. tuberculosis* (reviewed in [15]) have demonstrated that even though absence of IL-10 leads to better clearance of these pathogens, it can also result in severe and sometimes lethal pathologic changes. Therefore, although modulation of the IL-10 signaling pathway could be a potential target to avoid the establishment of chronic infection, more studies are needed to elucidate the optimal activation of the immune system necessary to improve clearance of chronic pathogens without a great cost to the host.

## Materials and Methods

### Bacterial strains, media and culture conditions

Bacterial strains used in this study were the virulent strain *Brucella abortus* 2308 and its isogenic mutant strain MX2 which has an insertion of pKSoriT-*bla*-*kan*-P*sojA*-*mCherry* plasmid [37]. For strain MX2, positive clones were kanamycin resistant and fluorescent, as previously described [37]. Strains were cultured on tryptic soy agar (Difco/Becton-Dickinson, Sparks, MD) or tryptic soy broth at 37°C on a rotary shaker. Bacterial inocula for mouse infection were cultured on tryptic soy agar plus 5% blood for 3 days [39]. For cultures of strain MX2, kanamycin (Km) was added to the culture medium at 100 µg/mL. All work with *B. abortus* cells was performed at biosafety level 3.

### Bone marrow derived macrophage infection

Bone marrow-derived macrophages were differentiated from bone marrow precursors from femora and tibiae of female, 6 to 8 weeks old, C57BL/6J and IL-10^−/−^ mice obtained from The Jackson Laboratory (Bar Harbor) following a previously published procedure [40]. For BMDM experiments, 24-well microtiter plates were seeded with macrophages at concentration of 5×10^5^ cells/well in 0.5 mL of RPMI media (Invitrogen, Grand Island, NY) supplemented with 10% FBS and 10 mM L-glutamine (RPMI supl) incubated for 48 h at 37°C in 5% CO_2_. Preparation of the inoculum and BMDM infection was performed as previously described [40]. Briefly, for inoculum preparation, *B. abortus* 2308 was grown for 24 h and then diluted in RPMI supl, and about 5×10^7^ bacteria in 0.5 mL of RPMI supl were added to each well of BMDM, reaching multiplicity of infection (MOI) of 100. Microtiter plates were centrifuged at 210× *g* for 5 min at room temperature in order to synchronize infection. Cells were incubated for 20 min at 37°C in 5% CO_2_, free bacteria were removed by three washes with phosphate-buffered saline (PBS), and the zero-time-point sample was taken as described below. After the PBS wash, RMPI supl plus 50 mg gentamicin per mL was added to the wells, and the cells were incubated at 37°C in 5% CO_2_. For cytokine production assays, supernatants from each well were sampled at 0, 8, 24, or 48 h after infection, depending on the experiment performed. In order to determine bacterial survival, the medium was aspirated at the time points described above, and the BMDM were lysed with 0.5 mL of 0.5% Tween 20, followed by rinsing of each well with 0.5 mL of PBS. Viable bacteria were quantified by serial dilution in sterile PBS and plating on TSA. For gene expression assays, BMDM were resuspended in 0.5 mL of TRI-reagent (Molecular Research Center, Cincinnati) at the time-points described above and kept at −80°C until further use. When necessary, 1 ng/mL of mouse rIL-10 (eBioscience, San Diego, CA) or 1 ng/mL of mouse rIFN-γ (BD Bioscience, San Jose, CA) was added to the wells and kept throughout the experiments. All experiments were performed independently in triplicate at least three times and the standard error for each time point calculated.

### RAW-Blue macrophages experiments

RAW-Blue cells (Invivogen, San Diego, CA) were derived from RAW-264.7 macrophages with chromosomal integration of a SEAP reporter construct inducible by NF-κB and AP-1. RAW-Blue cells were maintained in Zeocin (Invivogen, San Diego, CA) selective medium. For RAW-Blue experiments, 24-well microtiter plates were seeded with macrophages at concentration of 2×10^5^ cells/well in 0.5 mL of DMEM media (Invitrogen, Grand Island, NY) supplemented with 10% FBS and 10 mM L-glutamine (DMEM supl). Preparation of the inoculum and RAW-Blue infection was performed as previously described [40], using MOI = 100. For NF-κB activation assays, supernatant from each well was sampled at 8 h and 24 h after infection and secretion of the substrate SEAP was detected and measured in a spectrophotometer at 650 nm with QUANTI-Blue (Invivogen, San Diego, CA) according to manufacturer's instructions. When necessary, 1 ng/mL of mouse rIL-10 (eBioscience, San Diego, CA),1 ng/mL of mouse rIFN-γ (BD Bioscience, San Jose, CA),1 µg/mL of anti-mouse IL-10R antibody (R&D Systems, Minneapolis, MN) or anti-mouse IgG isotype antibody control (R&D Systems, Minneapolis, MN) were added to the wells and kept throughout the experiments. All experiments were performed independently in triplicate at least three times and the standard error for each time point calculated.

### Ethics statement

Experiments with mice were carried out in strict accordance with the recommendations in the Guide for Care and Use of Laboratory Animals of the National Institute of Health and were approved by the Institutional Animal Care and Use Committees at the University of California at Davis (protocol number: 16468).

### Animal experiments

Female C57BL/6J wild-type mice, B6.129P2*-Il10^tm1Cgn^/J*; (IL-10^−/−^) mice [29] and *Il-10* GFP reporter mice [19], aged 6–8 weeks, were obtained from The Jackson Laboratory (Bar Harbor). Female and male *Il10^flox/flox^Cd4cre^+/−^* (IL-10flox/CD4Cre), and *Il10R^flox/flox^Lysmcre^+/−^* (IL-10Rflox/LysMCre) aged 6–8 weeks, were reported previously [27], [31]. Female and male *IL10^flox/flox^LysMCre^+/−^* (IL-10flox/LysMCre) were generated at UC Davis. For the strains IL-10 flox/CD4Cre, IL-10Rflox/LysMCre and IL-10flox/LysMCre, littermate *Il10^flox/flox^Cd4cre^−/−^, Il10R^flox/flox^Lysmcre^−/−^*, and *IL10^flox/flox^LysMCre^−/−^* mice were used as control, respectively. Mice were held in microisolator cages with sterile bedding and irradiated feed in a biosafety level 3 laboratory. Groups of 3 to 5 mice were inoculated intraperitoneally (i.p.) with 0.2 mL of phosphate-buffered saline (PBS) containing 5×10^5^ CFU of *B. abortus* 2308 as previously described [41]. At 3, 9, 15, 21 and/or 42 days after infection, depending on the experiment performed, the mice were euthanized by CO_2_ asphyxiation and their serum, livers and spleens were collected aseptically at necropsy. The livers and spleens were homogenized in 2 mL of PBS, and serial dilutions of the homogenate were plated on TSA for enumeration of CFU. Samples of liver and spleen tissue were also collected for gene expression and histopathology analysis as described below.

### ELISA

The presence of IL-10, IL-6 and TNF-α in BMDM supernatant and in serum samples from C57BL/6, IL-10flox/CD4Cre, IL-10flox/LysMCre and littermate control mice infected with *B. abortus* 2308 was determined by indirect enzyme-linked immunosorbent assay (ELISA) (eBioscience, San Diego, CA) according to the manufacturer's instructions. The ELISA test was read at 450 nm with an ELISA microplate reader (MR5000; Dynatech). The sensitivity of the ELISA used was 7.8 pg/mL. Data points are the averages of duplicate dilutions, with each measurement being performed twice.

### RT-PCR and real time PCR analysis

Eukaryotic gene expression was determined by real-time PCR as previously described [40]. Briefly, eukaryotic RNA was isolated using TRI reagent (Molecular Research Center, Cincinnati) according to the manufacturer's instructions. A Reverse transcriptase reaction was performed to prepare complementary DNA (cDNA) using TaqMan reverse transcription reagents (Applied Biosystems, Carlsbad). A volume of 4 µL of cDNA was used as template for each real-time PCR reaction in a total reaction volume of 25 µL. Real-time PCR was performed using SYBR-Green (Applied Biosystems) along with the primers listed in Table S1 in Text S1. Data were analyzed using the comparative Ct method (Applied Biosystems, Carlsbad). Transcript levels of *Il10*, *Il6*, *Ifng* and *Tnfa* were normalized to mRNA levels of the housekeeping gene *βactin*.

### Histopathology

Formalin fixed spleen and liver tissue sections were stained with hematoxylin and eosin, and two veterinary pathologists (MX and TS) performed a blinded evaluation using criteria described in Table S2 in Text S1. Representative images were obtained using an Olympus BX41 microscope and the brightness adjusted (Adobe Photoshop CS2).

### Flow cytometry

Flow cytometric analysis of IL-10 producing cells was performed in splenocytes from IL-10 GFP reporter mice infected for 3 and 9 days with *B. abortus* 2308. Briefly, after passing the spleen cells through a 100-µm cell strainer and treating the samples with ACK buffer (0.15 M NH_4_Cl, 1.0 mM KHCO_3_, 0.1 mM Na_2_EDTA [pH 7.2]) to lyse red blood cells, splenocytes were washed with PBS (Gibco) containing 1% bovine serum albumin (fluorescence-activated cell sorter [FACS] buffer). After cell counting, 4×10^6^ cells/mouse were re-suspended in PBS and stained with Aqua Live/Dead cell discriminator (Invitrogen, Grand Island, NY) according to the manufacturer's protocol. After Live/Dead staining, splenocytes were resuspended in 50 µL of FACS buffer and cells were stained with a cocktail of anti-B220 Brilliant Violet 421 (Biolegend, San Diego, CA), anti-CD3 PE (BD Pharmingen, San Jose, CA), anti-CD11b APC.Cy7 (Biolegend, San Diego, CA), anti-F4/80 Pe.Cy7 (Biolegend, San Diego, CA), anti-Cd11c APC (Biolegend, San Diego, CA). To determine the T cell subset responsible for IL-10 production, cells were stained with a cocktail of anti-CD3 APC.Cy7 (eBioscience, San Diego, CA), anti-CD8 AF700 (BD Pharmigen, San Jose, CA), anti-TCRγδ PE (BD Pharmigen, San Jose, CA), anti-CD4 eFluor 450 (eBioscience, San Diego, CA), anti-CD25 Pe.Cy7 (eBioscience, San Diego, CA). The cells were washed with FACS buffer and fixed with 4% formaldehyde for 30 min at 4°C, and resuspended in FACS buffer prior to analysis. Flow cytometry analysis was performed using an LSRII apparatus (Becton Dickinson, San Diego, CA), and data were collected for 5×10^5^ cells/mouse. Resulting data were analyzed using Flowjo software (Treestar, inc. Ashland, OR). Gates were based on Fluorescence-Minus-One (FMO) controls.

### Immunofluorescence microscopy

Immunofluorescence of *Brucella* infected BMDM was performed as previously described [24]. Briefly, *B. abortus* MX2 infected BMDM were grown on 12-mm glass coverslips in 24-well plates were washed three times with PBS, fixed with 3% paraformaldehyde, pH 7.4, at 37°C for 20 min, washed three times with PBS and then incubated for 10 min in 50 mm NH_4_Cl in PBS in order to quench free aldehyde groups. Samples were blocked and permeabilized in 10% goat serum and 0.1% saponin in PBS for 30 min at room temperature. Cells were labeled by inverting coverslips onto drops of primary antibodies diluted in 10% horse serum and 0.1% saponin in PBS and incubating for 45 min at room temperature. The primary antibody used was rat anti-mouse LAMP-1 (BD Pharmigen, San Jose, CA). Bound antibodies were detected by incubation with 1∶500 dilution of Alexa Fluor 488 donkey anti-rat (Invitrogen, Grand Island, NY) for 45 min at room temperature. Cells were washed twice with 0.1% saponin in PBS, once in PBS, once in H_2_O and then mounted in Mowiol 4-88 mounting medium (Calbiochem). Samples were observed on a Carl Zeiss LSM 510 confocal laser scanning microscope for image acquisition (Carl Zeiss Micro Imaging). Confocal images of 1024×1024 pixels were acquired as projections of three consecutive slices with a 0.38-µm step and assembled using Adobe Photoshop CS2 (Adobe Systems). For quantification of *Brucella* MX2 and Lamp1+ compartment colocalization, at least 100 bacteria/sample were counted. All experiments were performed independently in quadruplicate at least two times.

### Statistical analysis

Fold changes of ratios (bacterial numbers or mRNA levels) and percentages (flow cytometry and fluorescent microscopy) were transformed logarithmically prior to statistical analysis. An unpaired Student's *t*-test was performed on the transformed data to determine whether differences in fold changes between groups were statistically significant (P<0.05). Significance of differences in histopathology scores was determined by a one-tailed non-parametric test (Mann-Whitney).

## Supporting Information

Figure S1 Expansion of CD4^+^CD25^+^ T cells during *Brucella* infection. (**A**) Flow cytometry quantification of CD4^+^CD25^+^ splenic T cells from C57BL/6 IL-10 GFP-reporter mice infected with *B. abortus* 2308 for 9 days. (**B**) Representative data plot of CD4 and CD25 expression in splenic T cells from C57BL/6 IL-10 GFP-reporter mice infected with *B. abortus* 2308 for 9 days. Values represent mean ± SEM. *P<0.05. n = 4. Values represent mean ± SEM. (*) represents P<0.05 relative to uninfected control using unpaired t-test statistical analysis. FMO = fluorescence minus one.
(TIF)

Figure S2 (**A, B**) Histopathology score of spleen (**A**) and liver (**B**) from littermate mice (Cre^−/−^) compared with IL10flox/LysMCre mice (Cre^+/−^) at 3, 9 and 21 d.p.i. (**C**) Representative histopathology figures from (**A,B**) - Black arrows indicate microgranulomas, white arrowheads show neutrophilic infiltrate, and white upper case WP indicates white pulp (×20). n = 5. (*) represents P<0.05 using Mann-Whitney statistical analysis.
(TIF)

Figure S3 (**A**) ELISA essay for IL-10 production in serum from littermate control mice (grey bars) compared with IL10flox/CD4Cre mice (white bars) at 0, 3, 9, 21 and 42 d.p.i. (**B**) qRT-PCR analysis of IL-10 expression in spleen from littermate control (grey bars) compared with IL10flox/CD4Cre mice (white bars) at 3, 9, 21 and 42 d.p.i. (**C**) qRT-PCR analysis of pro-inflammatory cytokines genes (*Ifnγ*, *Il6* and *Tnfα*) in liver from littermate control (grey bars) compared with IL10flox/CD4Cre mice (white bars) at 3, 9, 21 and 42 d.p.i. n = 5. Values represent mean ± SEM. (*) represents P<0.05 relative to uninfected control using unpaired t-test statistical analysis.
(TIF)

Figure S4 (**A**) ELISA essay for IL-10 production in supernatant from C57BL/6 wild type BMDM infected with *B. abortus* 2308 for 24 h. (**B**) ELISA essay for IL-10 production in supernatant from RAW-Blue macrophages infected with *B. abortus* 2308 for 8 h and 24 h in the presence of IL-10 receptor blocking antibody (IL-10R Ab), isotype control (IgG Ab) or exogenous IL-10 (rIL-10).
(TIF)

Figure S5 qRT-PCR analysis of pro-inflammatory cytokines genes (*Ifnγ*, *Il6* and *Tnfα*) in liver from littermate control (grey bars) compared with IL10Rflox/LysMCre mice (white bars) at 3, 9, 21 and 42 d.p.i. n = 5. Values represent mean ± SEM. (*) represents P<0.05 relative to uninfected control using unpaired t-test statistical analysis.
(TIF)

Text S1 Supplementary Methods for real-time RT-PCR and histopathology scoring. Table S1: Real-time RT-PCR primers used in this study. Table S2: Histopathology scoring used in this study.
(DOCX)
