# Supplementary figures and images for: CD4+ T Cell-derived IL-10 Promotes Brucella abortus Persistence via Modulation of Macrophage Function
Source: PLoS Pathog. 2013 Jun 20;9(6):e1003454. doi: 10.1371/journal.ppat.1003454 (PMC3688575; doi:10.1371/journal.ppat.1003454)

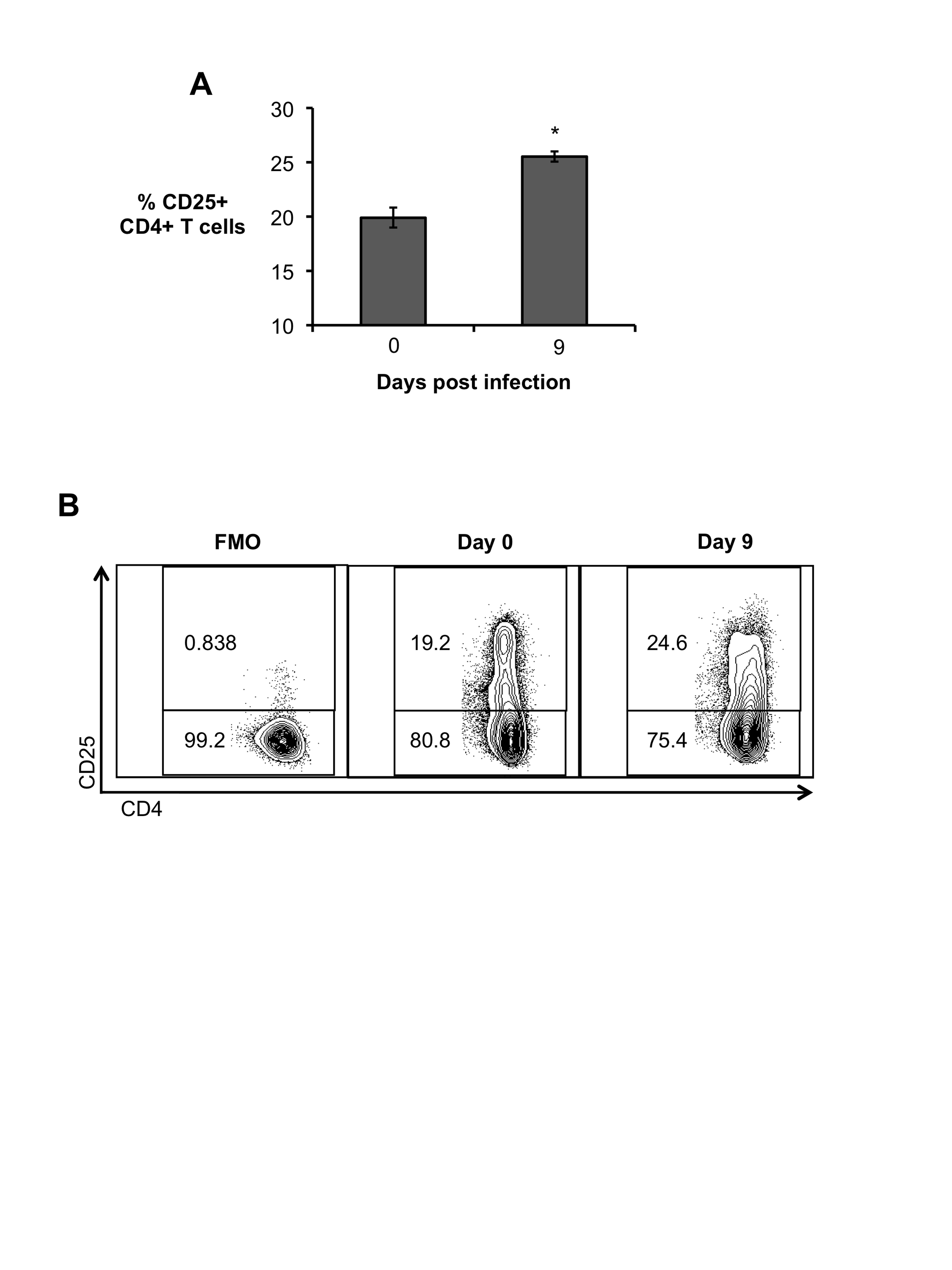

Supplement: Figure S1 — Expansion of CD4+CD25+ T cells during Brucella infection. (A) Flow cytometry quantification of CD4+CD25+ splenic T cells from C57BL/6 IL-10 GFP-reporter mice infected with B. abortus 2308 for 9 days. (B) Representative data plot of CD4 and CD25 expression in splenic T cells from C57BL/6 IL-10 GFP-reporter mice infected with B. abortus 2308 for 9 days. Values represent mean ± SEM. *P<0.05. n = 4. Values represent mean ± SEM. (*) represents P<0.05 relative to uninfected control using unpaired t-test statistical analysis. FMO = fluorescence minus one. (TIF) [file ppat.1003454.s001.tif]

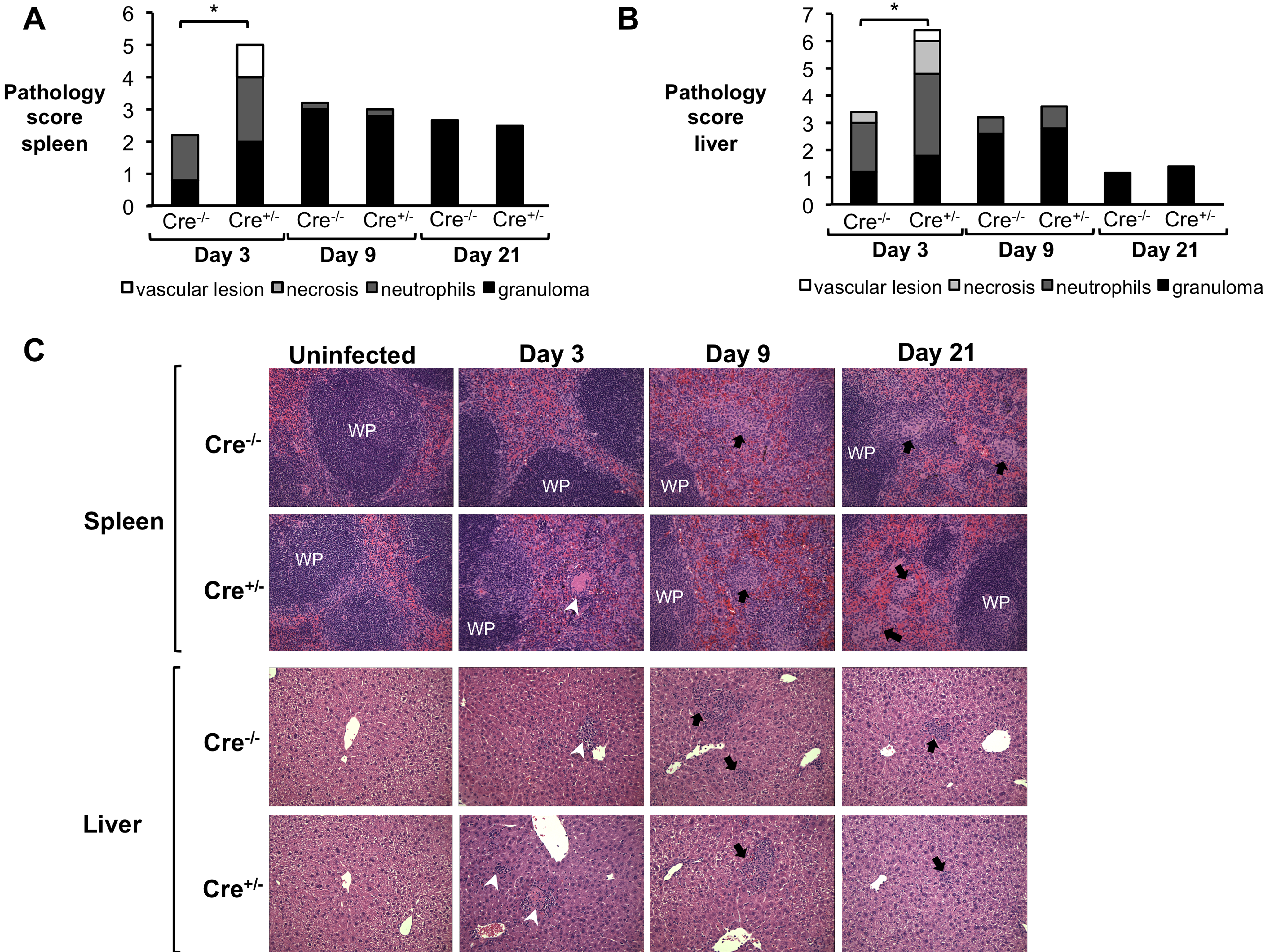

Supplement: Figure S2 — (A, B) Histopathology score of spleen (A) and liver (B) from littermate mice (Cre−/−) compared with IL10flox/LysMCre mice (Cre+/−) at 3, 9 and 21 d.p.i. (C) Representative histopathology figures from (A,B) - Black arrows indicate microgranulomas, white arrowheads show neutrophilic infiltrate, and white upper case WP indicates white pulp (×20). n = 5. (*) represents P<0.05 using Mann-Whitney statistical analysis. (TIF) [file ppat.1003454.s002.tif]

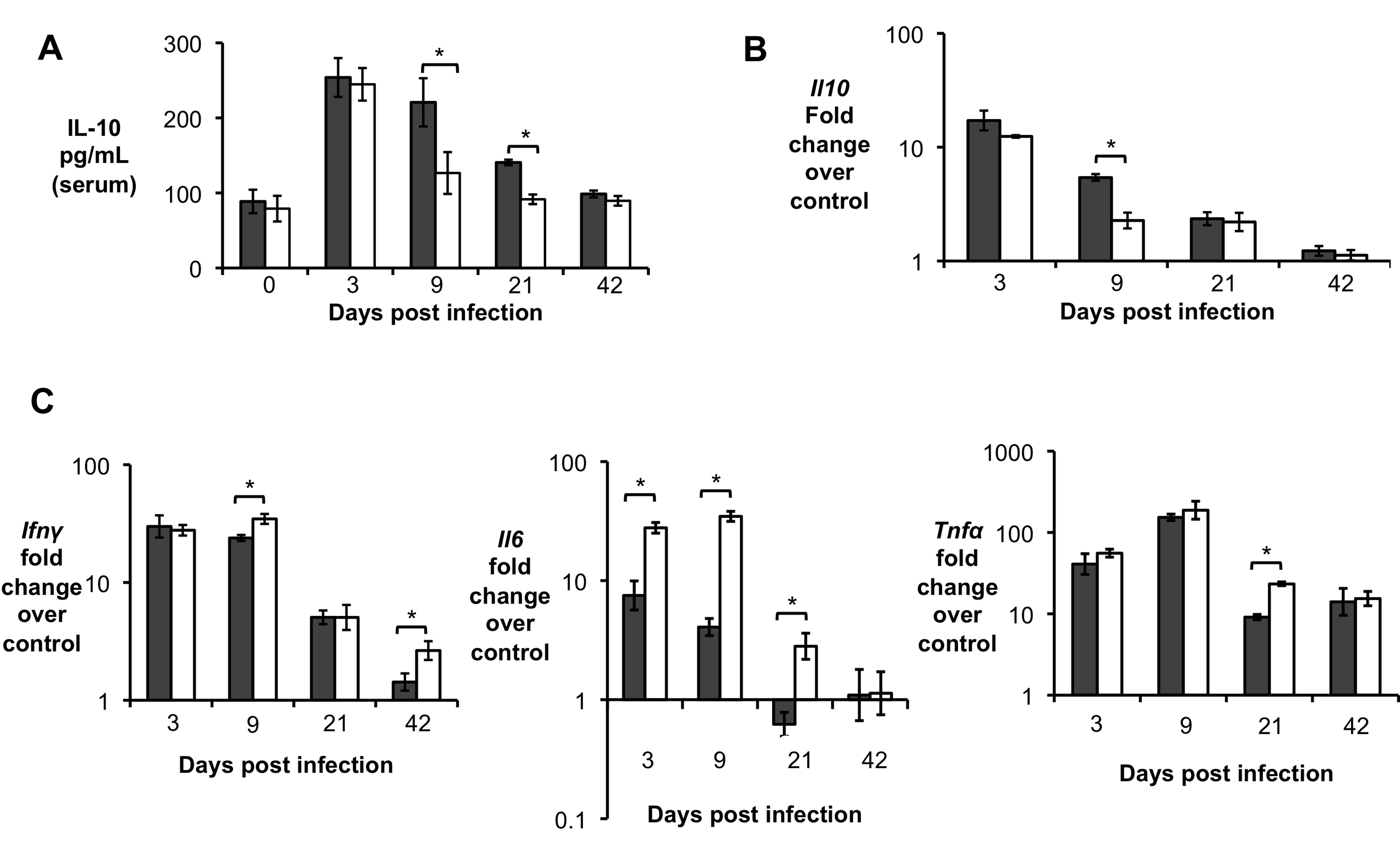

Supplement: Figure S3 — (A) ELISA essay for IL-10 production in serum from littermate control mice (grey bars) compared with IL10flox/CD4Cre mice (white bars) at 0, 3, 9, 21 and 42 d.p.i. (B) qRT-PCR analysis of IL-10 expression in spleen from littermate control (grey bars) compared with IL10flox/CD4Cre mice (white bars) at 3, 9, 21 and 42 d.p.i. (C) qRT-PCR analysis of pro-inflammatory cytokines genes (Ifnγ, Il6 and Tnfα) in liver from littermate control (grey bars) compared with IL10flox/CD4Cre mice (white bars) at 3, 9, 21 and 42 d.p.i. n = 5. Values represent mean ± SEM. (*) represents P<0.05 relative to uninfected control using unpaired t-test statistical analysis. (TIF) [file ppat.1003454.s003.tif]

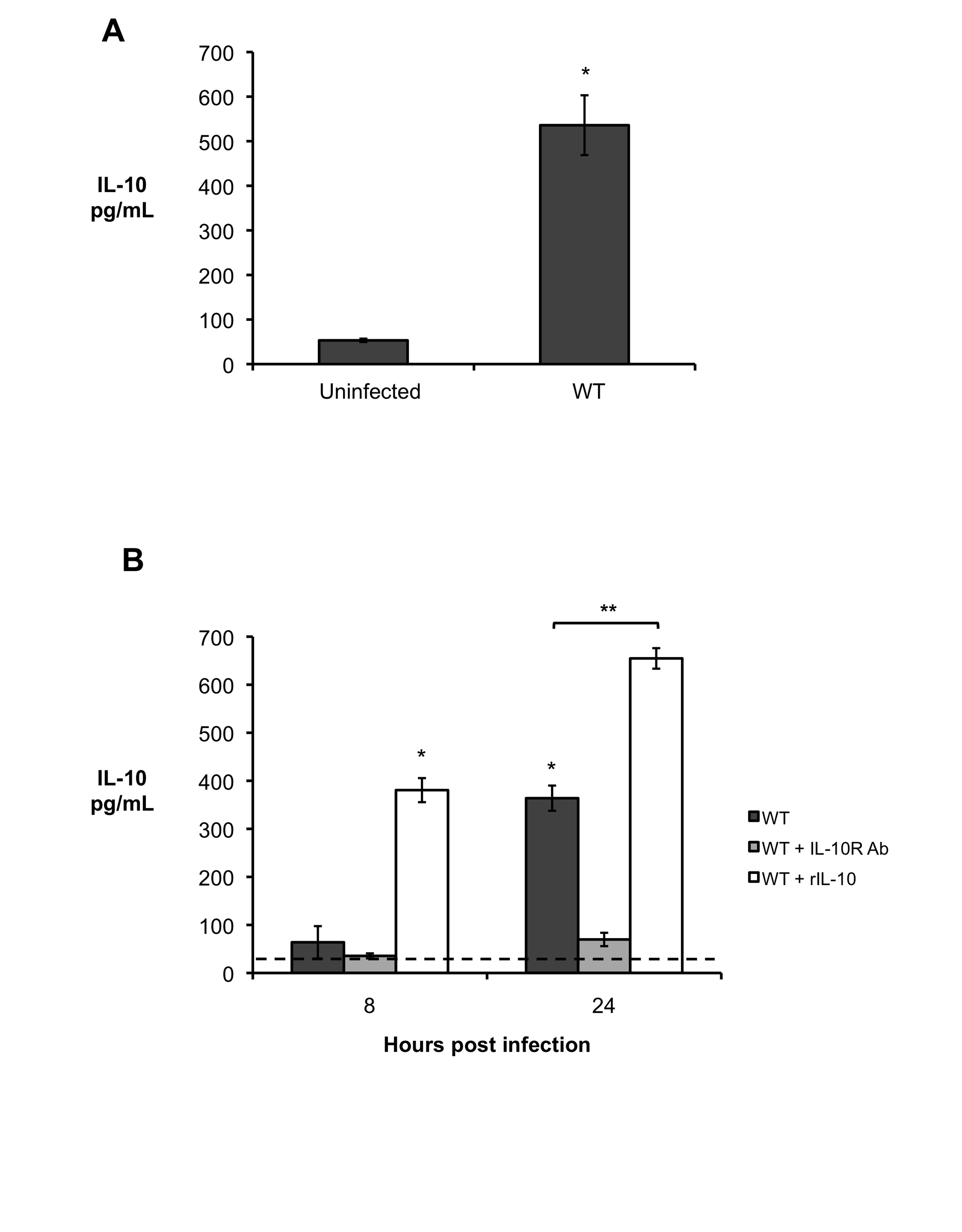

Supplement: Figure S4 — (A) ELISA essay for IL-10 production in supernatant from C57BL/6 wild type BMDM infected with B. abortus 2308 for 24 h. (B) ELISA essay for IL-10 production in supernatant from RAW-Blue macrophages infected with B. abortus 2308 for 8 h and 24 h in the presence of IL-10 receptor blocking antibody (IL-10R Ab), isotype control (IgG Ab) or exogenous IL-10 (rIL-10). (TIF) [file ppat.1003454.s004.tif]

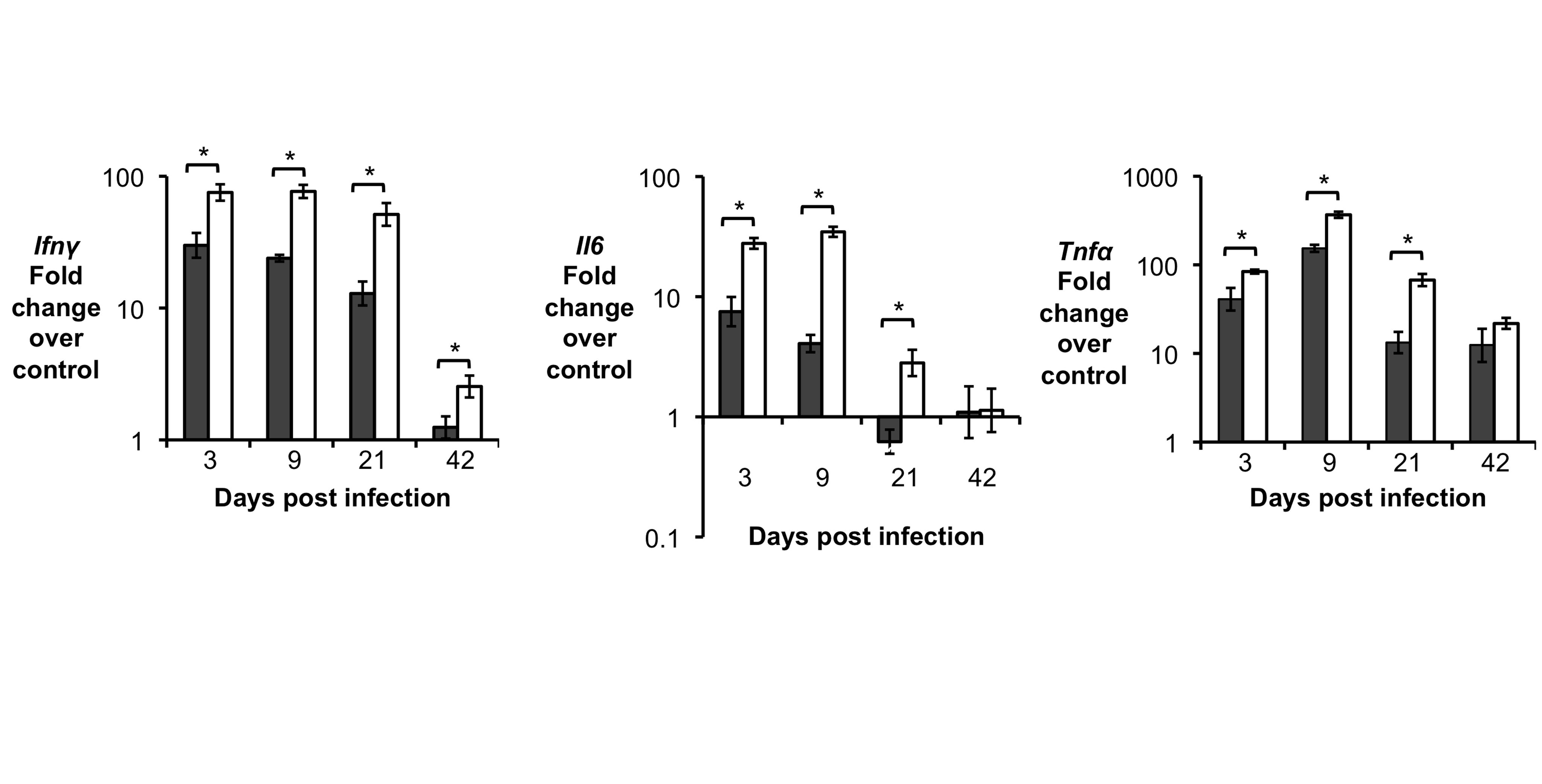

Supplement: Figure S5 — qRT-PCR analysis of pro-inflammatory cytokines genes (Ifnγ, Il6 and Tnfα) in liver from littermate control (grey bars) compared with IL10Rflox/LysMCre mice (white bars) at 3, 9, 21 and 42 d.p.i. n = 5. Values represent mean ± SEM. (*) represents P<0.05 relative to uninfected control using unpaired t-test statistical analysis. (TIF) [file ppat.1003454.s005.tif]
