# Supplementary material for: CD4+ T Cell-derived IL-10 Promotes Brucella abortus Persistence via Modulation of Macrophage Function
Source: PLoS Pathog. 2013 Jun 20;9(6):e1003454. doi: 10.1371/journal.ppat.1003454 (PMC3688575; doi:10.1371/journal.ppat.1003454)
Supplement: Text S1 — Supplementary Methods for real-time RT-PCR and histopathology scoring. Table S1: Real-time RT-PCR primers used in this study. Table S2: Histopathology scoring used in this study. (DOCX) [file ppat.1003454.s006.docx]

**SUPPORTING INFORMATION**

**Supplementary table 1.** Real-time PCR primers used in the present study:

| **Target gene** | **Sequence** |
| --- | --- |
| *β-actin* | FWD: 5'-AGAGGGAAATCGTGCGTGAC-3’ |
|  | REV: 5'-CAATAGTGATGACCTGGCCGT-3’ |
| *il10* | FWD: 5'-GGTTGCCAAGCCTTATCGGA-3’ |
|  | REV: 5'-ACCTGCTCCACTGCCTTGCT-3’ |
| *ifnγ* | FWD: 5'-TCAAGTGGCATAGATGTGGAAGAA-3’ |
|  | REV: 5'-TGGCTCTGCAGGATTTTCATG-3’ |
| *tnfα* | FWD: 5'-CATCTTGTCAAAATTCGAGTGACAA-3’ |
|  | REV: 5'-TGGGAGTAGACAAGGTACAACCC-3’ |
| *il6* | FWD: 5'-GCACAACTCTTTTCTCATTTCCACG-3’ |
|  | REV: 5'-GCCTTCCCTACTTCACAAGTCCG-3’ |

**Supplementary table 2.** Histopathology score used in the present study:

| **Score** | **Granuloma** | **Neutrophils** | **Necrosis** | **Vascular lesion** |
| --- | --- | --- | --- | --- |
| **0** | no lesion | no lesion | no lesion | no lesion |
| **1** | mild focal to multifocal granuloma formation | mild focal to multifocal neutrophilic infiltration | mild focal necrosis | mild focal vasculitis/thrombosis |
| **2** | mild to moderate focal to multifocal granuloma formation | mild to moderate muiltifocal neutrophilic infiltration | mild to moderate multifocal necrosis | mild to moderate multifocal vasculitis/thrombosis |
| **3** | moderate multifocal granuloma formation | moderate multifocal neutrophilic infiltration | moderate multifocal necrosis | moderate multifocal vasculitis/thrombosis |
| **4** | severe multifocal to coalescent granuloma formation | severe multifocal to coalescent neutrophilic infiltration | severe multifocal to coalescent necrosis | severe multifocal to diffuse vasculitis/thrombosis |
